# Nonadiabatic effects in electronic and nuclear dynamics

**DOI:** 10.1063/1.4996816

**Published:** 2018-01-09

**Authors:** Martin P. Bircher, Elisa Liberatore, Nicholas J. Browning, Sebastian Brickel, Cornelia Hofmann, Aurélien Patoz, Oliver T. Unke, Tomáš Zimmermann, Majed Chergui, Peter Hamm, Ursula Keller, Markus Meuwly, Hans-Jakob Woerner, Jiří Vaníček, Ursula Rothlisberger

**Affiliations:** 1 Laboratory of Computational Chemistry and Biochemistry, Institut des Sciences et Ingénierie Chimiques, Ecole Polytechnique Fédérale de Lausanne (EPFL), CH-1015 Lausanne, Switzerland; 2 Department of Chemistry, University of Basel, Klingelbergstrasse 80, CH-4056 Basel, Switzerland; 3 Physics Department, ETH Zurich, CH-8093 Zurich, Switzerland; 4 Laboratory of Theoretical Physical Chemistry, Institut des Sciences et Ingénierie Chimiques, Ecole Polytechnique Fédérale de Lausanne (EPFL), CH-1015 Lausanne, Switzerland; 5 Laboratoire de Spectroscopie Ultrarapide (LSU) and Lausanne Centre for Ultrafast Science (LACUS), Institut des Sciences et Ingénierie Chimiques, Ecole Polytechnique Fédérale de Lausanne (EPFL), CH-1015 Lausanne, Switzerland; 6 Department of Chemistry, University of Zurich, Zürich, Switzerland; 7 Laboratorium für Physikalische Chemie, ETH Zürich, CH-8093 Zürich, Switzerland

## Abstract

Due to their very nature, ultrafast phenomena are often accompanied by the occurrence of nonadiabatic effects. From a theoretical perspective, the treatment of nonadiabatic processes makes it necessary to go beyond the (quasi) static picture provided by the time-independent Schrödinger equation within the Born-Oppenheimer approximation and to find ways to tackle instead the full time-dependent electronic and nuclear quantum problem. In this review, we give an overview of different nonadiabatic processes that manifest themselves in electronic and nuclear dynamics ranging from the nonadiabatic phenomena taking place during tunnel ionization of atoms in strong laser fields to the radiationless relaxation through conical intersections and the nonadiabatic coupling of vibrational modes and discuss the computational approaches that have been developed to describe such phenomena. These methods range from the full solution of the combined nuclear-electronic quantum problem to a hierarchy of semiclassical approaches and even purely classical frameworks. The power of these simulation tools is illustrated by representative applications and the direct confrontation with experimental measurements performed in the National Centre of Competence for Molecular Ultrafast Science and Technology.

## I. INTRODUCTION

### A. Definition of adiabatic versus nonadiabatic processes

The terms adiabatic and nonadiabatic, respectively, are used in many different contexts. Whereas in classical thermodynamics, adiabatic refers to processes that occur without any exchange of heat or matter between a system and its surrounding, here, we will instead adopt a generic quantum mechanical definition of adiabaticity.

In 1928, Born and Fock formulated the adiabatic theorem according to which a physical system under the influence of a time-dependent perturbation remains in its instantaneous eigenstate if the perturbation is slow enough and the energy separation between the eigenvalue of the current eigenstate and the rest of the spectrum of the Hamiltonian is large enough.^1^ In other words, we will consider a process to be adiabatic if an applied time-dependent change is so slow that all the characteristic degrees of freedom of the system have enough time to adapt at every instant, i.e., the system reaches its equilibrium at all times and can be described with a single (stationary) eigenstate instead of a (time-dependent) superposition. One can also say that in the nonadiabatic regime, the perturbation induces coupling between the different eigenstates. It becomes already apparent from this definition that the two characteristic quantities that determine if a process is adiabatic or not are a separation of characteristic time and energy scales.

Let us consider a system with an intrinsic relaxation time characterized by the frequency *w^in^* (*in* for *intrinsic*) subject to a time-dependent perturbation with frequency *w^ex^* (*ex* for *external*). The system is currently in an eigenstate with eigenvalue *e_i_* of an entire possible spectrum of values *e_j_*. Then, adiabatic conditions will be met if *w^in^* ≫ *w^ex^* and $|e_{i}-e_{j}|\gg 0$.

In the following, we will discuss several examples of systems characterized by different types of degrees of freedom subject to various time-dependent changes and will try to establish connections between the distinct pictures.

We will first consider nonadiabatic effects occurring during tunnel ionization of single atoms or molecules in intense laser fields. Here, the characteristic degrees of freedom are associated with the electron dynamics and the time-dependent change is induced by the external laser field. If the laser field varies slowly enough, the electronic wavefunction can adapt instantaneously to the external field and the system remains in the adiabatic regime. The characteristic time/energy scales are quantified by the Keldysh parameter^2^

$$\gamma :=\frac{\sqrt{2I_{p}}}{E_{\text{max}}}\omega ,$$ (1)

where *I*_p_ is the ionization potential of the target, *E*_max_ is the peak electric field strength, and *ω* is the angular frequency of the applied laser.

Next, we will discuss nonadiabatic effects in the combined dynamics of electrons and nuclei. Here, the time-dependent change is caused by the variations of the electrostatic field generated by the movement of the positively charged nuclei and the characteristic separation of time and energy scales is the one between electronic and nuclear motion, *w^ele^* versus *w^nuc^*, that is also indirectly proportional to the relative masses of electrons with respect to nuclei, m^*ele*^ versus M^*nuc*^, and the energy separation of electronic eigenstates ΔE. The adiabatic regime is approached for *w^ele^* ≫ *w^nuc^* and ΔE ≫ 0. Under these conditions, the electronic and nuclear degrees of freedom can be fully separated and the time-evolution of the system can be described by solving the time-independent Schrödinger equation for every given set of fixed nuclei (clamped nuclei approximation) and thus the system evolves adiabatically on a single potential energy surface (PES) [Born-Oppenheimer (BO) approximation]. In regions where the characteristic time scales of electrons and nuclei become comparable and the energy gap between different potential energy surfaces becomes small such as in the vicinity of avoided crossings or conical intersections, the Born-Oppenheimer approximation breaks down and nonadiabatic effects quantified by the nonadiabatic couplings have to be taken into account.

As a third class of nonadiabatic phenomena, we will discuss the nonadiabatic coupling of vibrational modes. It has recently been shown^3^ that a similar approach to the one that is used in the treatment of electronic-nuclear nonadiabatic processes can also be employed to identify vibrational conical intersections between potential energy surfaces along specific vibrational modes.

The general structure of this review is as follows: we will first introduce the general theory in form of the Born-Oppenheimer approximation and the different representations that are used as a basis to represent the electronic states in the case of a breakdown of the BO, namely the adiabatic representation that is used for on-the-fly dynamics versus diabatic representations, in which the nuclear kinetic energy operator is diagonal and the coupling terms are scalar quantities that are numerically easier to evaluate. We also introduce the general formalism used to describe the quantum dynamics of a system in an electromagnetic field and we discuss different quantitative measures of nonadiabaticity. In the next chapter, we give a short summary of different quantum mechanical methods that are able to describe nonadiabatic effects starting with solutions of the full time-dependent electron-nuclear quantum problem through exact nonadiabatic quantum dynamics using the split-operator method necessarily limited to small systems in the gas phase, to different semiclassical methods (meanfield Ehrenfest dynamics and fewest switches surface hopping) to multisurface adiabatic reactive molecular dynamics (MS-ARMD) based on classical force fields (FF). This is followed by illustrative examples of joint experimental/theoretical studies of nonadiabatic systems of increasing complexity starting from single atoms, and molecules in the gas phase to many-atom systems in condensed phase.

This review thus covers a broad range of nonadiabatic phenomena from a variety of fields that often use this term in a different manner and apply other theoretical tools for the description of these processes. By discussing these approaches in a single review, we hope to help identifying pertinent differences, as well as common denominators.

## II. THEORY

### A. The Born-Oppenheimer approximation

#### 1. Derivation and origin

The behaviour of a closed, non-relativistic quantum system is completely characterised by the time-dependent Schrödinger equation (TDSE)

$$i\hbar \frac{d}{dt}\left|\Psi (t)\right\rangle =\hat{H}\left|\Psi (t)\right\rangle ,$$ (2)

where |Ψ⟩ is a state vector in Hilbert space. For a molecular system, the time-independent Hamiltonian ${\hat{H}}_{\text{mol}}$ is given by

$${\hat{H}}_{\text{mol}}=\sum_{\alpha }\frac{{\hat{P}}_{\alpha }^{2}}{2M_{\alpha }}+{\hat{H}}_{\text{el}},$$ (3)

$${\hat{H}}_{\text{el}}=\sum_{i}\frac{{\hat{p}}_{i}^{2}}{2}+\sum_{i<j}\frac{1}{{\hat{r}}_{\text{ij}}}-\sum_{\alpha ,i}\frac{Z_{\alpha }}{\left|{\hat{R}}_{\gamma }-{\hat{r}}_{i}\right|}+\sum_{\alpha <\beta }\frac{Z_{\alpha }Z_{\beta }}{{\hat{R}}_{\alpha \beta }}$$ (4)

$$={\hat{T}}_{\text{el}}+{\hat{V}}_{\text{ee}}+{\hat{V}}_{\text{eN}}+{\hat{V}}_{\text{NN}},$$ (5)

where the *electronic Hamiltonian* ${\hat{H}}_{\text{el}}$ has been introduced. The eigenvalues of the position operators $\hat{r}$ and $\hat{R}$ are the set of electronic and nuclear coordinates described by the collective variables {**r**} and {**R**}, respectively, while $\hat{p}$ and $\hat{P}$ denote the corresponding momentum operators. The molecular Hamiltonian contains kinetic terms due to the nuclei *α* and electrons *i*, the potential energy due to the interaction of electrons and nuclei (${\hat{V}}_{\text{eN}}$), as well as nuclear-nuclear (${\hat{V}}_{\text{NN}}$) and electron-electron (${\hat{V}}_{\text{ee}}$) repulsion terms.

The state vector encodes all the information on the system, but is by itself an abstract quantity. Its direct correspondence in real space, Ψ(**r**, **R**, *t*) depends both on nuclear and electronic coordinates and is, in this form, an untractable object for most systems of interest. If the molecular Hamiltonian were separable, a simplification could easily be achieved by a factorisation of the wavefunction into a nuclear and electronic component.^4,5^ However, due to the presence of ${\hat{V}}_{eN}$, the molecular Hamiltonian is not separable. The question on how to separate nuclear and electronic degrees of freedom has therefore been of paramount importance to molecular quantum mechanics, be it for static or dynamic approaches.

Indeed, a first ansatz to this problem was proposed by Born and Oppenheimer as early as 1927,^6^ and was later generalised by Born and Huang in 1954.^7^ The different time scales of electronic and nuclear motion lie at the very heart of their approach. A non-separable Hamiltonian may be written as the tensor product of two subsystems; in particular, ${\hat{H}}_{\text{mol}}={\hat{H}}_{\text{fast}}⊗{\hat{H}}_{\text{slow}}$, where one Hamiltonian is due to the fast motion of the electrons, and the other is due to the slower, nuclear components.^5^ The spectrum of ${\hat{H}}_{\text{fast}}$ can then be expanded in terms of electronic eigenstates |Φ⟩ by taking the limit of clamped (or “frozen”) nuclei, a limit in which the kinetic contribution of the nuclei vanishes. Only terms due to the electronic Hamiltonian remain, ${\hat{H}}_{\text{fast}}={\hat{H}}_{\text{el}}$; since the potential energy still contains the terms ${\hat{V}}_{\text{NN}}$ and ${\hat{V}}_{\text{eN}}$, the dependency of the Hamiltonian on the nuclear coordinates **R** becomes parametric. For any nuclear configuration **R**, one can therefore obtain a set of electronic eigenstates

$${\hat{H}}_{\text{el}}(R)\left|\Phi_{l};R\right\rangle =E_{l}(R)\left|\Phi_{l};R\right\rangle ,$$ (6)

where the eigenfunctions $\left|\Phi_{l};R\right\rangle$ depend parametrically on **R** through ${\hat{V}}_{eN}$. Since ${\hat{H}}_{\text{el}}$ and $\hat{R}$ commute, the basis of the molecular Hamiltonian ${\hat{H}}_{\text{mol}}$ can be constructed from the direct product of eigenfunctions of $\hat{R}$ and the eigenstates of the fast Hamiltonian,^5^ $\left|R,\Phi_{l};R\right\rangle =\left|R\right\rangle ⊗\left|\Phi_{l};R\right\rangle$. After introducing a resolution of identity in this basis into the real-space projection of the state vector, Ψ(r,R,t)=⟨r,R|Ψ(t)⟩, by orthogonality, one obtains a factorized expression for Ψ(**r**, **R**, *t*),

$$\Psi (r,R,t)=\langle r,R|\Psi (t)\rangle =\sum_{l}\left\langle r|\Phi_{l};R\rangle \langle R,\Phi_{l};R|\Psi (t)\right\rangle$$ (7)

$$=\sum_{l}\Phi_{l}(r;R)\chi_{l}(R,t).$$ (8)

Here, the nuclear components *χ_l_*(**R**, *t*) (or “nuclear wavefunctions”) are projections of the configurational basis onto the Hilbert space vector |Ψ(t)⟩, and the Φ_*l*_(**r**; **R**) are the electronic wavefunctions of a system with configuration {**R**}. Note that the Φ_*l*_(**r**; **R**) are time-independent. This is commonly referred to as the Born-Oppenheimer^6^ or Born-Huang^7^ ansatz for the total wavefunction.

The description of the nuclear dynamics of the system is obtained by inserting this ansatz into the time-dependent Schrödinger equation. The resulting coupled-channels equation describes the exact time-evolution of the nuclear dynamics

$$\begin{array}{c} i\hbar \frac{\partial }{\partial t}\chi_{k}(R,t)=\left[-\sum_{\alpha }\frac{\hbar^{2}}{2M_{\alpha }}\nabla_{\alpha }^{2}+E_{k}(R)\right]\chi_{k}(R,t)+\sum_{l}C_{kl}\chi_{l}(R,t), \end{array}$$ (9)

$$C_{kl}(R,t)=-\sum_{\alpha }\frac{\hbar^{2}}{2M_{\alpha }}D_{kl}^{\alpha }(R,t)+\sum_{\alpha }\frac{\hbar^{2}}{M_{\alpha }}d_{kl}^{\alpha }(R,t)\nabla_{\alpha }.$$ (10)

The terms collected under **C**_*kl*_(**R**, *t*) are mediating between different electronic states and are referred to as the *non-adiabatic coupling* terms, with the scalar quantity $D_{kl}^{\alpha }(R)$ being the *kinetic* coupling, and the vectorial quantity $d_{kl}^{\alpha }(R)$ being the *derivative* coupling

$$D_{kl}^{\alpha }(R)=\langle \Phi_{k};R|\nabla_{\alpha }^{2}\left|\Phi_{l};R\right\rangle ,$$ (11)

$$d_{kl}^{\alpha }(R)=\langle \Phi_{k};R|\nabla_{\alpha }\left|\Phi_{l};R\right\rangle .$$ (12)

These terms are responsible for part of the nuclear quantum effects.

In the *Born-Oppenheimer* (BO) approximation, these coupling terms are neglected by setting $D_{kl}^{\alpha }(R)=0$ and $d_{kl}^{\alpha }(R)=0$ (which is equivalent to neglecting the effect of the nuclear kinetic energy operator on the electronic wavefunction). This results in a simplified ansatz for the total wavefunction and a less complex nuclear dynamics

Ψ(r,R,t)=Φ(r;R)χ(R,t), (13)

$$i\hbar \frac{\partial }{\partial t}\chi (R,t)=\left[-\sum_{\alpha }\frac{\hbar^{2}}{2M_{\alpha }}\nabla_{\alpha }^{2}+E(R)\right]\chi (R,t).$$ (14)

This simplification is often justified by the fact that the coupling terms are small and weighted by the inverse of the (heavy) nuclear masses (which leads to the often stated simplification that the BO approximation separates nuclear from electronic degrees of freedom due to differences in mass). The limit of classical nuclei (point particles or delta functions centered at **R**) may be recovered by rewriting the nuclear components *χ*(**R**) in polar representation^8–10^

$$\chi (R,t)=A(R,t)\;\text{exp}\;\left[\frac{i}{\hbar }S(R,t)\right].$$ (15)

Insertion of the above expression (15) into Eq. (14), separating real and imaginary parts and taking the classical limit ℏ→0, results in an isomorphism with the classical Hamilton-Jacobi equation of motion

$$\frac{\partial S(R,t)}{\partial t}+\sum_{\alpha }\frac{1}{2M_{\alpha }}{\left(\nabla_{\alpha }S(R,t)\right)}^{2}+E(R)=0.$$ (16)

In BO dynamics, the nuclei evolve according to the forces due to a single electronic state. In the limit of classical nuclei, this results in delta functions being propagated on a single potential energy surface (PES), neglecting all nuclear quantum effects. Some of these nuclear quantum effects such as zero-point energy and tunneling may be recovered by resorting to techniques such as the path integral formalism.^11^

Still, in order to describe the complete array of nuclear quantum effects, the non-adiabatic coupling elements must imperatively be included, since any dynamics based on the BO formalism forbids the nuclei—described as classical point charges or nuclear wave packets—to switch between potential energy surfaces; BO dynamics is therefore a strict *single state* dynamics. For a more extensive review, see, e.g., Refs. 9, 10, 12, and 13

#### 2. Breakdown of the Born-Oppenheimer formalism

Born-Oppenheimer dynamics completely neglects the nonadiabatic coupling vectors, assuming they are small when compared to the remaining terms. It is possible to express first order scalar coupling terms in a convenient form that depends on the eigenvalues of ${\hat{H}}_{\text{el}}$,^14^

$$d_{kl}^{\alpha }(R)=\frac{\langle \Phi_{k};R|\nabla_{\alpha }{\hat{H}}_{\text{el}}\left|\Phi_{l};R\right\rangle }{E_{l}(R)-E_{k}(R)}.$$ (17)

This expression reveals that the nonadiabatic coupling terms become important whenever two eigenstates are close in energy. In the case of a conical intersection, where the eigenvalues of ${\hat{H}}_{\text{el}}$ are degenerate, they even diverge. In such systems, the Born-Oppenheimer approximation breaks down and with it, the picture of classical nuclei evolving on either of two potential energy surfaces is no longer suitable. Due to faster nuclear motion, the nuclear wavepacket cannot be thought of as being localised on one PES, but instead spreads over several electronic states. In order to properly describe the dynamics of those systems, it is therefore necessary to go beyond the BO and to include the nuclear quantum effects that lead to couplings between electronic states.

### B. Diabatic and adiabatic representations

If only the *S* lowest-lying electronic states participate in the dynamics of the molecule, the state of the molecule can be written as

$$\left|\psi (t)\right\rangle =\sum_{n=1}^{S}\left|\psi_{n}(t)\right\rangle \left|n\right\rangle =\left(\begin{array}{c} \psi_{1}(t)\\ \vdots \\ \psi_{S}(t) \end{array}\right),$$ (18)

where $\left|\psi_{n}(t)\right\rangle$ is a time-dependent nuclear wavepacket on the *n*th potential energy surface, and |n⟩ is the corresponding *n*th electronic state. In the absence of the electromagnetic field, evolution of |ψ(t)⟩ is given by the time-dependent Schrödinger equation

$$i\hbar \frac{d}{dt}\left|\psi (t)\right\rangle ={\hat{H}}_{0}\left|\psi (t)\right\rangle ,$$ (19)

with the time-independent molecular Hamiltonian ${\hat{H}}_{0}$. While there are many possible choices for the basis of electronic states, the most useful two are the diabatic and adiabatic bases.

In the *diabatic representation*, the electronic basis states are assumed to be independent of the nuclear coordinates; therefore, the molecular Hamiltonian can be written as

$${\hat{H}}_{0}:=\hat{T}\;1+{\hat{V}}_{0},$$ (20)

where $\hat{T}$ is the nuclear kinetic energy operator

$$\hat{T}:=\frac{1}{2}{\hat{P}}^{T}\cdot M^{-1}\cdot \hat{P},$$ (21)

$M\equiv \text{diag}(M_{1},M_{2},\ldots ,M_{D})$ denotes the diagonal nuclear mass matrix and $\hat{P}$ the *D*-component vector of nuclear momenta. The second component of ${\hat{H}}_{0}$ is the molecular diabatic potential energy ${\hat{V}}_{0}\equiv V_{0}(\hat{Q})$, which depends on the *D* nuclear coordinates *Q*. The diabatic potential energy is diagonal at the reference geometry *Q*_0_, but away from this reference geometry, its off-diagonal elements, called *diabatic couplings*, are nonzero, and lead to transitions between diabatic electronic states.

By diagonalizing the potential energy matrix in the diabatic representation, one obtains the *adiabatic representation*, in which the molecular Hamiltonian is given by

$${\hat{H}}_{0}:=\frac{1}{2}{\left(\hat{P}1-i\hbar \hat{F}\right)}^{T}\cdot M^{-1}\cdot \left(\hat{P}1-i\hbar \hat{F}\right)+\hat{E},$$ (22)

where $\hat{E}\equiv E(Q)\equiv \text{diag}\left(E_{1}(Q),\ldots ,E_{S}(Q)\right)$ is a diagonal matrix of adiabatic potential energy surfaces and $\hat{F}\equiv F(Q)$ stands for (a *D*-component vector consisting of *S *×* S* matrices of) *nonadiabatic couplings*, which arise from the dependence of the adiabatic electronic basis states on nuclear coordinates

$$F_{jk}(Q):=\left\langle j(Q)|\nabla k(Q)\right\rangle ,$$ (23)

and which give rise to electronic transitions between adiabatic states.

Above, we have introduced a compact notation in which the boldface denotes the *S*-component vectors in the electronic Hilbert space, or *S *×* S* matrices representing electronic operators, whereas the hat ^ denotes nuclear operators.

### C. Quantum dynamics of a molecule interacting with an electromagnetic field

In the presence of electromagnetic field, evolution of |ψ(t)⟩ is given by the time-dependent Schrödinger equation (TDSE)

$$i\hbar \frac{d}{dt}\left|\psi (t)\right\rangle =\hat{H}(t)\left|\psi (t)\right\rangle ,$$ (24)

driven by the Hamiltonian

$$\hat{H}(t):={\hat{H}}_{0}+{\hat{V}}_{\text{int}}(t),$$ (25)

consisting of the molecular Hamiltonian ${\hat{H}}_{0}$ and the time-dependent potential ${\hat{V}}_{\text{int}}(t)$ representing the interaction of the molecule with the field. Within the electric dipole approximation,^15^ the interaction potential is given by

$${\hat{V}}_{\text{int}}(t)=-\hat{\vec{\mu }}\cdot \vec{E}(t),$$ (26)

where $\hat{\vec{\mu }}$ is the molecular electric dipole operator, $\vec{E}(t)$ is the electric field, and the arrow indicates three-dimensional vectors.

### D. Quantifying nonadiabaticity

**FIG. 1.**
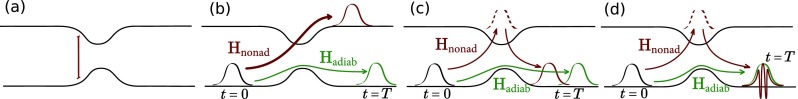
Criteria of nonadiabaticity of quantum dynamics. (a) The static energy-gap criterion does not take into account the dynamics of the wave packet. (b) The population transfer criterion measures the actual decay of probability density on the initial surface. It is more sensitive than the energy-gap criterion. “Adiabaticity,” i.e., the fidelity (overlap) of the adiabatic and exact wavefunctions can capture the population transfer (b) as well as other nonadiabatic effects such as displacement (c) or interference (d) on a single surface, which would be undetected by the population transfer criterion. **H**_adiab_ is the decoupled adiabatic Born-Oppenheimer Hamiltonian, whereas **H**_nonad_ is the fully coupled nonadiabatic Hamiltonian. Adapted with permission from Zimmermann and Vaníček, J. Chem. Phys. **136**, 094106 (2012). Copyright 2012 AIP Publishing LLC.

There are various ways in which one can quantify the importance of nonadiabatic effects on the dynamics. The simplest criteria rely on the “static” properties of the nonadiabatic Hamiltonian such as the strength of nonadiabatic couplings or the size of energy gaps between potential energy surfaces [Fig. 1(a)]. While useful as a starting point, such criteria do not take into account the dynamics and thus may sometimes yield an entirely wrong answer. More reliable are criteria which account for the motion of the wave packet, the simplest among such criteria being the population dynamics [Fig. 1(b)]. While the population decay of the initial electronic state is a clear sign of the importance of nonadiabatic effects, as shown in Ref. 16, the nonadiabatic effects may be important even in the absence of population decay. For example, the nonadiabatic wave packet may be displaced with respect to the adiabatic wave packet [Fig. 1(c)], or it may acquire additional quantum phase [Fig. 1(d)]. This is often accompanied by a transient population decay but there exist remarkable examples in which the population remains constant for all times, whereas the overlap of the adiabatic and nonadiabatic wavepackets decays quickly to zero.^17,18^

Motivated by these shortcomings, Zimmermann and Vaníček,^16–18^ and MacKenzie *et al.*^19^ proposed to quantify the importance of nonadiabatic couplings by defining the *adiabaticity* as the squared overlap (or *fidelity*)

$$F(t):={\left|f(t)\right|}^{2}:={\left|\left\langle \psi_{\text{adiab}}(t)|\psi_{\text{nonad}}(t)\right\rangle \right|}^{2},$$ (27)

between the wave function $\psi_{\text{nonad}}(t)$ evolved with the full molecular Hamiltonian ${\hat{H}}_{\text{nonad}}\equiv {\hat{H}}_{0}$ and the corresponding wavefunction $\psi_{\text{adiab}}(t)$ evolved with a diagonal Hamiltonian ${\hat{H}}_{\text{adiab}}\equiv \frac{1}{2}{\hat{P}}^{T}\cdot M^{-1}\cdot \hat{P}+\hat{E}$. Obviously, when *F*(*t*) ≈ 1, the dynamics is approximately adiabatic, whereas if *F*(*t*) ≈ 0, dynamics is strongly nonadiabatic (see Fig. 1).

In the above form, the definition applies in the adiabatic basis in the absence of the electromagnetic field. In the presence of the electromagnetic field, one would simply include the full interaction potential ${\hat{V}}_{\text{int}}(t)$ in ${\hat{H}}_{\text{nonad}}$ and only the diagonal terms of ${\hat{V}}_{\text{int}}(t)$ (those that induce rovibrational transitions only) to the ${\hat{H}}_{\text{adiab}}$. In the diabatic representation, one may define *diabaticity*^17^ in an analogous fashion as the fidelity, i.e., the squared overlap between the wavefunctions evolved with the full Hamiltonian and the Hamiltonian ignoring the diabatic couplings.

**FIG. 2.**
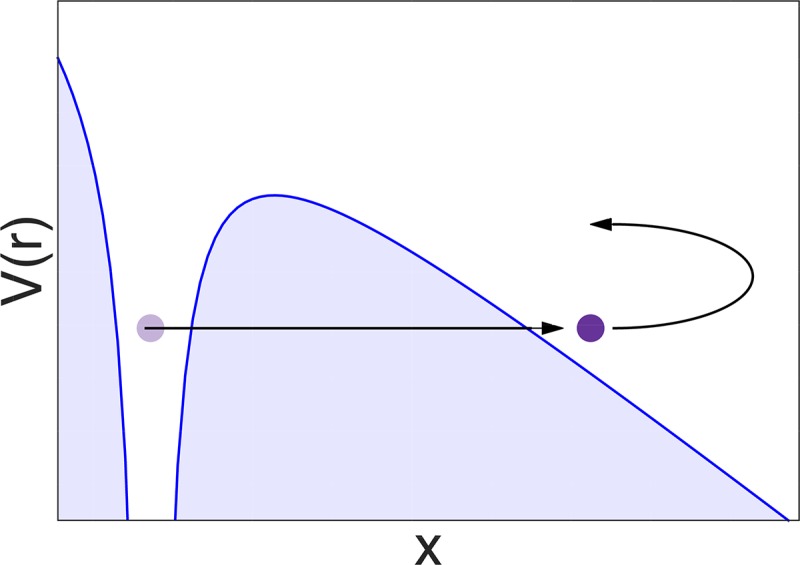
Quasistatic picture of strong-field tunnel ionization.

As it turns out, the adiabaticity can be estimated very efficiently with the semiclassical multiple surface dephasing representation (MSDR)^16–18^ which, among other things, allows to determine how many coupled electronic states must be included in a rigorous nonadiabatic quantum simulation. The MSDR evaluates the adiabaticity, or, more generally, quantum fidelity amplitude *f*(*t*), approximately as

$$f_{\text{MSDR}}\left(t\right)=h^{-D}{\text{Tr}}_{e}\int d^{2D}X\rho_{W}^{\text{init}}\left(X\right)\cdot Te^{-i\int_{0}^{t}V_{W}^{I}\left(X,t^{\prime }\right)dt^{\prime }/\hbar },$$ (28)

where *D* is the number of nuclear degrees of freedom, *X* ≡ (*Q*, *P*) denotes the 2*D* phase space coordinates of the nuclei, subscript W stands for the Wigner transform, $\rho_{W}^{\text{init}}$ is the Wigner transform of the density operator of the initial state, Tr_*e*_ is the trace over electronic degrees of freedom, and ${\hat{V}}^{I}\left(t\right)$ represents the nonadiabatic couplings in the interaction picture.

The computational cost of the MSDR scales favorably with the number of degrees of freedom and despite its semiclassical origin the method does not require the Hessians of the potential energy surfaces. Surprisingly, an explicit nonadiabatic simulation is not needed (although it may be used); instead, *f*_MSDR_ can be estimated by performing a simpler adiabatic simulation. In addition to evaluating the importance of nonadiabatic couplings, after only a slight modification, the MSDR can be used to evaluate the accuracy of an approximate nonadiabatic Hamiltonian.^16^

## III. METHODS FOR NONADIABATIC DYNAMICS

The theoretical description of nonadiabatic processes is a highly active field of research and a variety of methods have been developed to treat such phenomena.^20–33^

### A. Exact nonadiabatic dynamics using the split-operator method

In the absence of the electromagnetic field, the molecular state evolves as

$$\left|\psi (t)\right\rangle =\text{exp}\;(-i{\hat{H}}_{0}t/\hbar )\left|\psi (0)\right\rangle .$$ (29)

Note that this “free” evolution can be useful even when the dynamics is induced by electromagnetic field, on condition that the field is weak, and one may employ the first-order time-dependent perturbation theory (i.e., when the linear response theory is valid). When the electromagnetic field is present and cannot be treated perturbatively, the *exact* evolution of the molecular state is given by

$$\left|\psi (t)\right\rangle =T\;\text{exp}\left[-i\int_{0}^{t}\hat{H}(t^{\prime })dt^{\prime }/\hbar \right]\left|\psi (0)\right\rangle ,$$ (30)

where T denotes the time-ordering operator.^34^

There are various ways to implement these two formal solutions. Particularly appealing are so-called geometric integrators, which preserve the Lie group structure of the exact quantum evolution, such as its unitarity, time-reversibility, and symplecticity.^35^

An example of a geometric integrator is the split-operator algorithm,^36,37^ which, in general, only applies in the diabatic basis since it requires the Hamiltonian to be separable into a sum of two terms, one of which depends only on the momenta, and the other only on the coordinates. This is satisfied by the molecular Hamiltonian ${\hat{H}}_{0}$ in the diabatic representation [Eq. (20)], but not by ${\hat{H}}_{0}$ in the adiabatic representation [Eq. (22)], as the latter contains the products of the nonadiabatic couplings, which are functions of nuclear coordinates, with nuclear momenta. The basic idea is to first decompose the full evolution into a number of small time steps, since then, for small enough time step *t*, the evolution of the molecular state can be approximated to the second order as^36^

$$\begin{array}{c} \;\text{exp}\;(-i{\hat{H}}_{0}t/\hbar )=\text{exp}\;(-i\hat{T}t/2\hbar )\;\text{exp}(-i{\hat{V}}_{0}t/\hbar )\;\text{exp}(-i\hat{T}t/2\hbar )+O(t^{3}), \end{array}$$ (31)

in the absence of the electromagnetic field, and as^38^

$$\begin{array}{c} \;\text{exp}\;\left[-i\int_{0}^{t}\hat{H}(t^{\prime })dt^{\prime }/\hbar \right]=\text{exp}\;(-i\hat{T}t/2\hbar )\;\text{exp}\;(-i\hat{V}(t/2)t/\hbar )\text{exp}\;(-i\hat{T}t/2\hbar )+O(t^{3}), \end{array}$$ (32)

when the field is present. Note that in Eq. (32), the full potential energy $\hat{V}(t):={\hat{V}}_{0}+{\hat{V}}_{\text{int}}(t)$ is evaluated at the midpoint *t*/2. The last two expressions suggest that the quantum state can be easily evolved alternately, either with the kinetic energy $\hat{T}$, which is done trivially in the momentum representation, where $\text{exp}\;(-i\hat{T}t/\hbar )$ is diagonal, or with the potential energy [${\hat{V}}_{0}$ or $\hat{V}(t)$], which, in turn, is almost trivial in the coordinate representation. Note that in the case of nonadiabatic dynamics, there is a small modification to the standard, adiabatic split-operator algorithm: while the propagation is diagonal in either coordinate or momentum representation, one must perform an exponential of an *S *×* S* matrix **V**(*Q*, *t*) at each nuclear coordinate *Q*, in order to account for the transitions between electronic states. Curiously, the most expensive part of the propagation turns out to be the switching between the coordinate and momentum representations with the fast Fourier transform algorithm. While we have implemented these integrators with an arbitrary order of accuracy in the time step,^39,40^ here, for simplicity, we have restricted the presentation to the standard, second-order algorithms.

### B. Semiclassical methods

#### 1. Ehrenfest dynamics

An alternative possibility to include the effects of several electronic states within a semiclassical approach is based on an ansatz for the total wavefunction of

$$\Psi (r,R;t)=\Phi (r;t)\chi (R;t)\;\text{exp}\;\left[-\frac{i}{\hbar }\int_{t_{0}}^{t}E_{el}(t\prime )dt\prime \right],$$ (33)

$$E_{\text{el}}(t)=\iint \Phi^{*}(r,t)\chi^{*}(R,t){\hat{H}}_{\text{el}}(r,R)\chi (R,t)\Phi (r,t)\;dR\;dr.$$ (34)

This single-configuration ansatz gives rise to *Ehrenfest dynamics.*^9^ In contrast to the BO formalism, the time-dependent electronic wavefunction Φ(**r**; *t*) exhibits no dependence on the nuclear coordinates at all, not even parametrically. Instead, an additional exponential term, the *phase term* is introduced. By inserting this ansatz into the TDSE, the evolution of the nuclear and electronic wavefunctions is given by the Time-Dependent Self-Consistent Field (TDSCF) equations^41^

$$i\hbar \frac{\partial }{\partial t}\Phi (r,t)=\left[{\hat{T}}_{\text{el}}+\int dR\;\chi^{*}(R)\hat{V}(r,R)\chi (R)\right]\Phi (r,t),$$ (35)

$$i\hbar \frac{\partial }{\partial t}\chi (R,t)=\left[{\hat{T}}_{N}+\int dr\;\Phi^{*}(r,t){\hat{H}}_{\text{el}}(r,R)\Phi (r,t)\right]\chi (R,t),$$ (36)

where $\hat{V}={\hat{V}}_{\text{ee}}+{\hat{V}}_{\text{eN}}+{\hat{V}}_{\text{NN}}$. The nuclei evolve in a time-dependent *mean field* of the electronic states, and vice versa. As described above for the Born-Oppenheimer ansatz, the limit of classical nuclei can be recovered by inserting the polar representation of the nuclear wavefunction, (15), into the time-derivative of Eq. (36) and taking the limit ℏ→0.^9^ The resulting equation is isomorphic to the Hamilton-Jacobi equation and may be further recast to yield a Newton-like equation for the nuclei

$$-F_{\alpha }(t)=\nabla_{R_{\alpha }}\int dr\;\Phi^{*}(r,t){\hat{H}}_{\text{el}}(r,R)\Phi (r,t).$$ (37)

According to (37), the classical nuclei evolve on a single PES due to Φ(**r**, *t*). The corresponding equation for the electronic degrees of freedom is obtained by writing $\chi (R,t)=\sum_{\alpha }\delta (R(t)-R_{\alpha }(t))$; it is simply the time-dependent Schrödinger equation for Φ, with a (re)introduced parametric dependence on **R**,

$$i\hbar \frac{\partial \Phi (r;R(t),t)}{\partial t}={\hat{H}}_{\text{el}}(r,R)\Phi (r;R(t),t).$$ (38)

Therefore, the method lends itself to be combined with the time-dependent density functional theory (TD-DFT),^42^ where Φ in (37) can be expanded in terms of time-dependent Kohn-Sham non-interacting Slater determinants $\Phi (r,t)=\text{det}\left|\psi_{1}(r,t),\ldots ,\psi_{N}(r,t)\right|$, and **F**_*α*_ is derived using the Hellmann-Feynman theorem. Equations (37) and (38) have to be solved simultaneously, which can be carried out on-the-fly, e.g., by using a Runge-Kutta integrator.^42^

Due to the averaged nature of Φ(**r**, *t*), Ehrenfest dynamics is a suitable choice whenever the classical trajectories due to different electronic states do not differ considerably; such as when the relaxation of the electronic degrees of freedom is fast with respect to the nuclear motion. Otherwise, e.g., for molecular dissociations, the mean-field approximation may introduce large errors: After leaving regions of strong non-adiabaticity, the nuclei are unable to collapse on either of the PES, making their dynamics potentially unphysical.^9^ Ehrenfest dynamics is therefore typically limited to ultrafast or instantaneous processes.

#### 2. Trajectory surface hopping (TSH)

Trajectory Surface Hopping (TSH)^43^ offers another possibility of including nonadiabatic effects by independently propagating a swarm of particles on different, single electronic states, and allowing them to “hop” onto a different state according to some predefined hopping probability. As in the case of Ehrenfest dynamics, this propagation can be carried out on-the-fly.

Given a set of initial nuclear coordinates and velocities, trajectories are propagated on a single electronic state according to the Born-Oppenheimer scheme. Subsequently, the probability for a jump is calculated. In a first approximation, the Landau-Zener^44,45^ transition probability may be used. Originally formulated in a diabatic framework, some simplifications allow for it to be reformulated based on the adiabatic quantities^10^

$$P(t)\approx \;\text{exp}\;\left(-\frac{\pi }{2\hbar }\frac{\text{min}\left\{{\left|\Delta E_{01}(t)\right|}^{2}\right\}}{\max\left\{\frac{\partial \left|\Delta E_{01}(t)\right|}{\partial t}\right\}}\right).$$ (39)

Here, *P* is the probability to perform a nonadiabatic transition at an avoided crossing for a two-state system and Δ*E*_01_ is the adiabatic electronic gap.

A more rigorous approach to the transition probabilities is given by Tully's “Fewest Switches” formulation of TSH.^46^ A set of complex amplitudes {*C*(*t*)} is assigned to every trajectory to quantify the degree of nonadiabadicity during the propagation. The complex amplitudes themselves evolve along each trajectory according to

$$i\hbar \frac{\partial C_{j}(t)}{\partial t}=\sum_{i}^{\infty }C_{i}(t)\left[E_{i}^{\text{el}}(R)\delta_{ij}-i\hbar \sigma_{ij}(R,t)\right],$$ (40)

where $\sigma_{ij}(R,t)=d_{ij}(R)\cdot \dot{R}$ and **d**_*ij*_(**R**) is the derivative coupling as defined in Eq. (12). After integration, the probability of hopping from state *j* to state *i* within an infinitesimal time interval d*t* is computed

$$g_{ij}(t,t+dt)=2\int_{t}^{t+dt}d\tau \frac{-ℜ\left[C_{i}(\tau )C_{j}^{*}(\tau )\sigma_{ij}\right]}{C_{j}(\tau )C_{j}^{*}(\tau )}.$$ (41)

The hop is then accepted or rejected according to a Metropolis criterion by comparing the hopping probability to a random number *ζ* ∈ [0, 1],

$$\sum_{k\leq i-1}g_{jk}<\zeta <\sum_{k\leq i}g_{jk}.$$ (42)

Total energy conservation is ensured by rescaling the nuclear velocities after an accepted hop. The trajectory *γ* is now further propagated along the new electronic state *i* in an adiabatic fashion, until the next hopping attempt.

The terms needed in trajectory surface hopping can be rigorously derived from the linear response time-dependent density functional theory (LR-TDDFT).^47^ The sum in Eq. (40) may be truncated after *N* adiabatic states, and the (diagonal) electronic eigenvalues $E_{i}^{\text{el}}$ may be replaced by a relative term, ${\tilde{V}}_{ij}=\left[E_{i}^{\text{el}}-E_{0}^{\text{el}}\right]\delta_{ij}$. ${\tilde{V}}_{ij}$ has an analogue in the TDDFT excitation energies *ω_i_*, and Eq. (40) may be recast in terms of set of transformed coefficients $\left\{\tilde{C}(t)\right\}$ and the TDDFT excitation energies {*ω*},

$$i\hbar \frac{\partial {\tilde{C}}_{i}(t)}{\partial t}={\tilde{C}}_{i}(t)\omega_{i}-i\hbar \sum_{j}^{N}{\tilde{C}}_{j}(t)\sigma_{ij}(R,t).$$ (43)

The *σ_ij_* are accessible *via* finite differences

$$\begin{array}{c} {\sigma_{ij}(R,t)|}_{t+dt/2}=\frac{1}{2dt}[\int dr\;\Phi_{i}^{*}(r;R(t))\Phi_{j}(r;R(t+dt))-\int dr\;\Phi_{i}^{*}(r;R(t+dt))\Phi_{j}(r;R(t))]. \end{array}$$ (44)

After integration of Eq. (40), the switching probability is computed according to Eq. (42), using ${\tilde{C}}_{j}$ rather than *C_j_* and by linearly interpolating *σ_ij_*(*τ*). This approach has been implemented in combination with a plane-wave pseudo-potential formalism.^47,48^

The nuclear dynamics in TSH are still governed by a single-state Born-Oppenheimer formalism, but the propagation of the complex amplitudes and the calculation of the hopping probability allow for a transition between states. Since TSH is carried out for a collection {*γ*} of trajectories, this hopping can mimick the spread of the wavepacket in non-adiabatic regions. However, nuclear quantum effects such as tunneling and the zero-point energy are not described, due to the classical nature of the individual trajectories.

### C. Fully classical methods

#### 1. Multisurface adiabatic reactive molecular dynamics (MS-ARMD)

Following a chemical reaction to determine the reaction pathway and to predict experimental observables is a key task for computational methods, since most atomistic aspects cannot be determined experimentally.^49^ Using quantum methods is usually inexpedient for large systems for the simple reason that the computational requirements are too high. Hence, alternative approaches, like Quantum Mechanics/Molecular Mechanics (QM/MM)^50^ and reactive force fields (FF) have been developed. The former, however, cannot be utilized to calculate converged reaction rates, due to the high computational cost of calculating hundreds of long-time trajectories. Therefore accurate reactive FF approaches offer the best means for sampling processes of interest in a statistically convincing approach.

Among other methods, such as the work by Warshel (Empirical Valence Bond (EVB)),^51^ Westmoreland (RMDff),^52^ van Duin and Goddard (ReaxFF),^53^ and Meuwly and co-workers developed multisurface adiabatic reactive molecular dynamics (MS-ARMD).^54–56^ This method, which aims to achieve quantum method accuracy with, in principle, the computational cost of conventional FF, couples (at least) two non-reactive, empirical FFs by means of a time- or energy-independent switching function *w_i_*. MS-ARMD is a widely applicable technique that can describe several different reaction pathways and conserves total energy. It was implemented in the widely used CHARMM program.^57^

In MS-ARMD adiabatic dynamics takes place on the lowest potential energy surface (PES) *V*_min_, as long as the energy of the higher lying surface(s) is not within a specified, user defined Δ*V* of energy. Surface-crossing is achieved by mixing the PESs through energy-dependent weights. Δ*V* acts as energy damping parameter that balances the individual weights with respect to the energy separation (“gap”) between the PESs. Products of Gaussian and polynomial functions (GAPOs), which depend on the energy difference between two surfaces (Δ*V_ij_*), are introduced for the purpose of reproducing the energy barrier from electronic structure calculations. The global, reactive PES is therefore described by

$$\begin{array}{c} V_{\text{MS}-\text{ARMD}}(x)=\sum_{i=1}^{n}w_{i}(x)V_{i}(x)+\sum_{i=1}^{n-1}\sum_{j=i+1}^{n}\left[w_{i}(x)+w_{j}(x)\right]\sum_{k=1}^{n_{ij}}\Delta V_{\text{GAPO},k}^{ij}(x), \end{array}$$ (45)

where *w_i_*(*x*) are normalised weights obtained from raw weights *w_i_*_,0_(*x*), calculated by $w_{i,0}(x)=\text{exp}(-(V_{i}(x)-V_{\text{min}})/\Delta V),\;V_{i}(x)$ is the energy of state *i*, and $\Delta V_{\text{GAPO},k}^{ij}(x)$ is the energy of the GAPO between state *i* and *j*. Evaluating energies and forces in MS-ARMD scales linearly with number of low lying surfaces. The time liming factor in this approach is fitting of a suitably parameterized PES.

## IV. ILLUSTRATIONS: EXPERIMENT AND THEORY

### A. Nonadiabatic effects in electron dynamics

#### 1. Time-dependent potentials due to external strong laser fields

Ultrafast lasers with a pulse duration of a few femtoseconds (fs = 10^−15 ^s) can reach peak intensities of the order of 10^13^ up to 10^15^ W/cm^2^. In these kind of regimes, the electric field of the laser pulse can compete with the binding Coulomb potential of an atom (or a molecule), and lead to ionization phenomena which go beyond the photoelectric effect. These phenomena allow the study of electron dynamics in their natural time scale of attoseconds (as = 10^−18 ^s).^58^ The first order perturbation theory describing the single photon absorption is insufficient to describe strong-field ionization. The strong field must be described as an oscillating field inducing temporal changes in the binding potential of the target system.

Therefore, the two key time scales in these phenomena are the periodicity of the laser field, and the typical response time of a bound electron wave function.^59^ The most often used laser source for strong-field experiments is a titanium-sapphire laser^60^ with a central wavelength of ≈800 nm, corresponding to a period of 2.7 fs ≈ 110 au. Most experiments are performed on noble gases to study the single atom response to strong fields. The typical response time of a bound electron wave function in an atom can be estimated as 1/*I*_p_ au for noble gases.^59^ Another time scale to compare is the duration for an electron to rotate once around it is core in the Bohr's atomic model, at the Bohr radius *a*_0_ distance from the core, which is ≈ 6.3 au ≈ 150 as. Both the response time as well as the Bohr rotation period are about 2 orders of magnitude shorter than the laser period. Throughout this section, atomic units “au” ($\hbar =m_{e}=e=a_{0}=1$) are employed unless specified otherwise.

#### 2. Concepts

The basis for the theoretical description of strong-field ionization was published by Keldysh about 50 years ago.^2^ In strong-field ionization, tree main regimes can be distinguished, based on their value of the Keldysh parameter^2^

$$\gamma :=\frac{\sqrt{2I_{p}}}{E_{\text{max}}}\omega ,$$ (46)

where *I*_p_ is the ionization potential of the target, *E*_max_ is the peak electric field strength, and *ω* is the angular frequency of the applied laser field. For *γ* ≪ 1, the mathematical limit of *γ* → 0 can be taken, which corresponds to neglecting the temporal dependence of the field, and describing the system in a quasistatic picture. For any time of interest, the shape of the combined potential is frozen, and the reaction of the electron wave function to a static situation is calculated. Therefore, the limit of *γ* ≪ 1 is the adiabatic approximation of strong-field ionization.^59,61,62^

In this adiabatic approximation of strong-field ionization, a bound electron can tunnel-ionize through the potential barrier created by the superposition of the Coulomb field and the electric field of the laser pulse, see Fig. 2. It is assumed that the temporal change of the potential is so slow that the bound wave function can always instantaneously adapt to the potential and always stays in equilibrium, following the general idea of an adiabatic approximation. This has several consequences, such as the fact that a tunnelling electron wave packet does not gain any energy, since the oscillation of the electric field is neglected and therefore the wave packet cannot absorb any photons (or they would have ≈0 energy due to the *γ* → 0 limit). Also, in the quasistatic picture, the tunnelling wave function cannot distinguish between a linearly polarized laser pulse, or an elliptical or circular laser pulse.

**FIG. 3.**
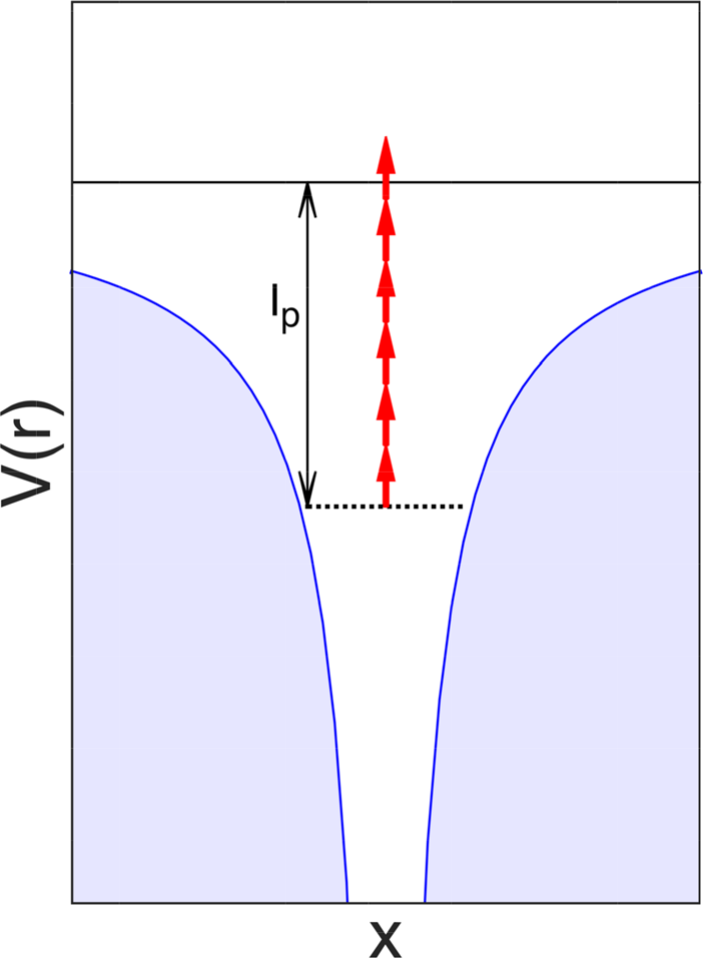
Multiphoton ionization for the case of *γ* ≫ 1.

The other extreme limit of the Keldysh parameter is *γ* ≫ 1, and describes the regime of multiphoton ionization, see Fig. 3. In this case, the field strength is not strong enough anymore to create a short enough potential barrier to allow tunnel ionization, and the absorption of multiple photons in order to gain enough energy to escape becomes comparatively more feasible.^63^

**FIG. 4.**
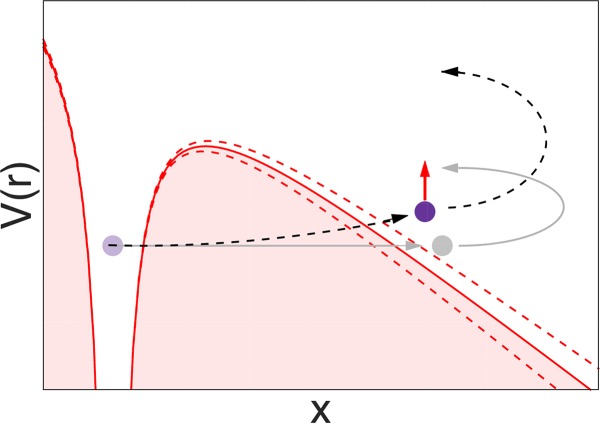
Nonadiabatic picture of strong-field tunnel ionization: the adiabatic approximation is shown in grey for comparison. For the nonadiabatic case, we take into account the dynamics of the laser field during the tunneling process, shown here with the dashed lines of the potential barrier. This allows the electron to gain energy through photon absorption which leads to a shorter exit radius (Ref. 87, Fig. 3 and Ref. 97) For elliptical polarization, the most probable electron trajectory exhibits a transverse momentum at the tunnel exit, tangential to the laser field rotation in the polarization plane (shown as a red arrow).^65^

The third main regime is the intermediate *γ* ∼ 1. In this case, the main ionization mechanism is still tunnelling through a potential barrier. However, the electron wave packet is not necessarily in equilibrium all the time, it feels the temporal change of the potential, and reacts to the dynamics of the system. Figure 4 illustrates this nonadiabatic regime of tunnel ionization.

**FIG. 5.**
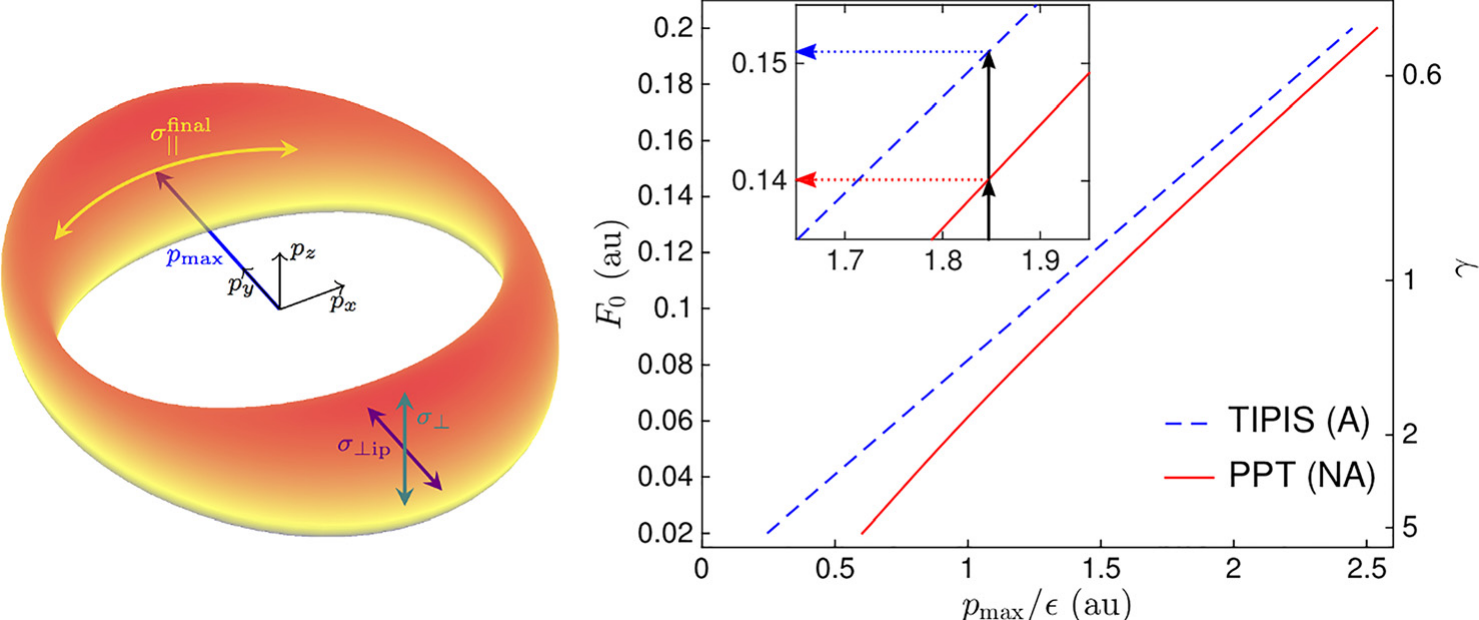
Left: asymptotic photoelectron momentum distribution for elliptically polarized ionizing field (major axis of polarization along *x*, minor axis along *y*). Adapted from Ref. 95. Right: prediction of *p*_max_ in the adiabatic (blue dashed) or nonadiabatic (red solid) framework, depending on the peak field strength. Reproduced with permission from Hofmann *et al.*, Phys. Rev. A **90**, 043406 (2014). Copyright 2014 American Physical Society.

#### 3. Methods for nonadiabatic dynamics

Analytical descriptions of strong-field ionization can be sorted into either the adiabatic or nonadiabatic framework, depending on whether they take the *γ* → 0 mathematical limit or not during the derivation. The most well known nonadiabatic formalism was derived by Perelomov, Popov, and Terent'ev (PPT) in 1966.^64,65^ The PPT theory predicts a laser cycle averaged ionization rate and the photoelectron momentum distribution after the laser pulse has passed. Based on PPT, many more recent formalisms have been developed, in an effort to reduce the number of included approximations, for example, Refs. 66–71. Common to all of these are three core approximations. The dipole approximation states that the spatial dependence of the laser field can be neglected since the wavelength is much larger than the target size, and the influence of magnetic fields via Lorentz force is negligible. Within the single active electron approximation, only the ionization of one electron is considered, and the other bound electrons are assumed to remain in their ground state. The strong field approximation states that the influence of the ion Coulomb field is neglected for the dynamics of the wave packet after ionization.

To complement analytical descriptions, different numerical approaches have been developed. The most commonly used are the numerical solution of the time-dependent Schrödinger equation (TDSE) and Classical Trajectory Monte Carlo (CTMC) simulations.^72^ But also the time-dependent density functional theory (TDDFT) is sometimes employed, particularly when the target to be ionized becomes larger.^73^

The TDSE solution (see, for example, Refs. 74–80) is naturally nonadiabatic, and uses a pseudopotential to approximate the Coulomb potential. Typically the pseudopotential is for a single active electron, though efforts have been put into developing methods that can take account of the correlation between two electrons to some degree.^81^ However, the computational effort for TDSE solutions is enormous, because both high spatial resolution and a large spatial box to evaluate the TDSE are necessary, and most often there are no symmetries in the system to reduce the dimensionality of the calculation.

CTMC simulations make use of analytical descriptions of the tunnel ionization process, and treat only the propagation of ionized photoelectrons in the continuum numerically.^82–85^ The initial conditions are chosen to replicate the probability distribution of the wave function at the tunnel exit, as calculated by the chosen analytical formalism. Consequently, CTMC simulations can be located in either the adiabatic or nonadiabatic regime, and suffer from the same approximations as the analytical descriptions, with the exception that the Coulomb force of the ion can be included in the classical Newton's equation of motion. It is also possible to take account of electron correlation during the propagation.^86^

#### 4. Tunnel ionization delays measured with the attoclock

Quantum tunnelling is very fundamental and sparked a long-standing debate on the time duration of this process.^87^ The attoclock is an angular streaking approach for the extraction of tunnelling delay time in the context of strong field ionization.^88,89^ The most recent attoclock measurements^90^ sparked a number of developments^65,78,81,91–97^ and showed that non-adiabatic effects are significant, but do not contradict finite tunnelling time. Taking into account all non-adiabatic effects, the values of the extracted tunnelling delay times are comparable to the results published in Ref. 90 although they are shifted to slightly lower field strengths.

Taking account of nonadiabatic effects in tunnel ionization leads to several changes in the observable photoelectron momentum spectrum. The probability of ionization is slightly less critically dependent on the instantaneous field strength, which leads to a broadening in the temporal distribution of ionization events.^67^ The predicted momentum distribution widths are larger in the nonadiabatic framework compared to the adiabatic approximation.^64–66,68^ The tunnelling wave packet notices a rotation of the field in case of elliptical or circular polarisation, gaining a momentum tangential to the rotation of the field until the tunnel exit.^65,66,69^ This leads to overall larger absolute values of photoelectron momenta after the laser pulse has passed.^95^ Another effect can be seen in the sketch of Fig. 4, the exit point of the tunnelling wave packet shifts closer to the atomic core, thanks to some energy gain during the tunnelling process.^65,66^

Since the peak field strength experienced by the targets must be calibrated *a posteriori* from the measured photoelectron momentum distribution (or energy spectrum)^79^ in strong-field ionization, experimentally showing the influence of nonadiabatic effects has proven difficult. Even contradicting conclusions have been reached based on the same experimental data, see, for example, Refs. 78 and 98 and 95, 99, and 100. The average absolute momentum *p*_max_ of photoelectrons in (highly elliptical or circularly) polarized strong fields is the observable of choice for the field strength calibration.^101^ However, this particular observable is affected by the transverse momentum imparted on the tunnelling wave packed in the nonadiabatic framework, leading to large differences in the calibrated field strength for the identical data (compare Fig. 5 right), solely depending on the theoretical description that was used in the calibration. To circumvent this issue, TDSE calculations are the preferred method.^79,95^ They are more reliable for small systems such as hydrogen or helium, because less approximations are necessary to describe the atomic target with the bound and ionized electron wave function. The commonly employed pseudopotentials completely neglect electron correlation, or even just a possible polarization of the remaining ion. Nevertheless, recently presented studies confirm that nonadiabatic effects are important to take account of.^78,95,102^

**FIG. 6.**
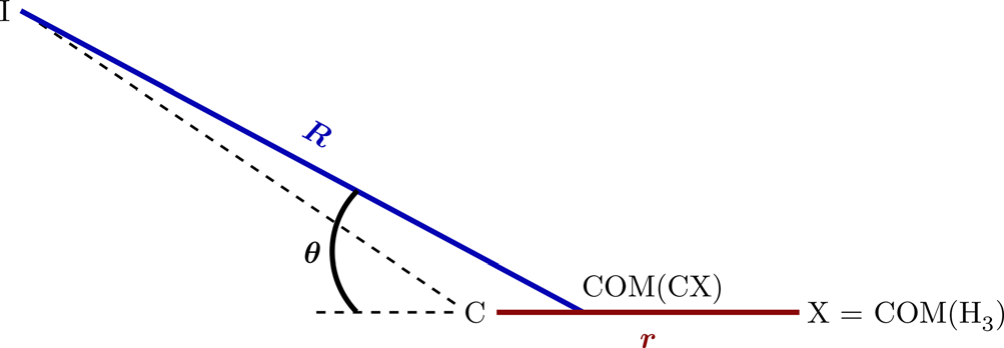
Jacobi coordinates used in the three-dimensional model of iodomethane.

### B. Nonadiabatic effects in combined electron-nuclear dynamics

#### 1. Photodissociation of iodomethane studied with exact nonadiabatic quantum dynamics

Photodissociation dynamics of iodomethane following the excitation to the A band has been studied since the discovery of the first laser relying on photodissociation and more recently by Wörner and co-workers^103–106^ with time-resolved high-harmonic spectroscopy. These methods were originally applied only to the electronic ground state of molecules, yet, ultrafast dynamics occurs predominantly in excited electronic states, and therefore Wörner *et al.* extended the methodology to excited states. First, two synchronized pump pulses are used to generate an intensity grating that induces a spatial modulation of the excited state's population. Then, using an intense femtosecond probe pulse, an electronic wave packet can be extracted from one of the valence orbitals and driven back to interfere with the remaining bound electronic state. If the electron recombines, extreme ultraviolet radiation is emitted. This phenomenon, known as high-harmonic generation, makes it possible to image a molecular orbital, probe vibrational dynamics, or observe a chemical reaction in real time.

The observed high-harmonic signal in the iodomethane experiment depends crucially on the population dynamics. To understand it better, we performed exact nonadiabatic quantum dynamics simulations of the photodissociation process induced by the pump pulse using reduced dimensionality models of iodomethane. These models allowed us to study systematically the importance of nonadiabatic dynamics and of various degrees of freedom in the dissociation process. Up to three active degrees of freedom, represented by the Jacobi coordinates (*R*, *r*, *θ*) (see Fig. 6) were used.^107^ The one- and two-dimensional models, considered only the *R* and (*R*, *r*) coordinates, respectively, and all three models were obtained by reducing the full nine-dimensional diabatic potential energy surfaces provided in Refs. 108 and 109 for the three electronic states of interest: A11,Q30+, and Q11. (The discarded coordinates were held fixed at their equilibrium values.) The nonadiabatic simulations required transition dipole moments coupling the electronic state A11 with Q30+, and nonadiabatic couplings coupling the electronic state Q30+ with Q11.

**FIG. 7.**
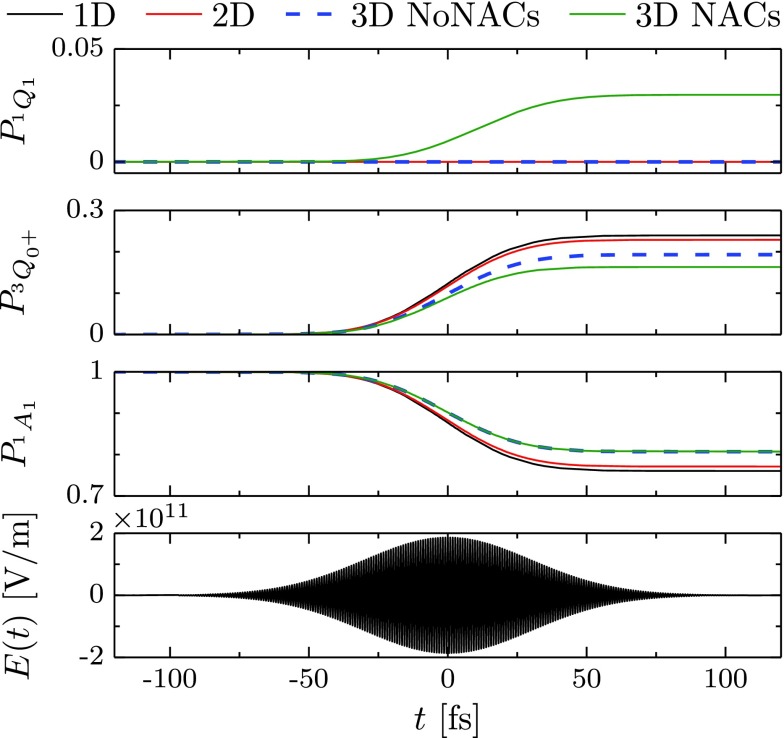
Population dynamics of iodomethane induced by an excitation with a pump laser pulse shown in the bottom panel. Time dependence of the populations of three electronic states in the different models: symbols 1D, 2F, and 3D stand for one-, two-, and three-dimensional models, while the acronyms NoNACs and NACs denote, respectively, models without and with nonadiabatic couplings.

The time-dependent Schrödinger equation with explicit time-dependent potential given by the electric dipole interaction with the laser field was solved by a generalization of the split-operator algorithm, which, in its standard form, requires that the Hamiltonian can be split into a position- and momentum-dependent parts.^38,39,110^ This is possible in the one- and two-dimensional models, containing only radial coordinates, which can be separated from the corresponding momenta simply by factoring out the radial dependence from the wavefunction, thus converting the radial coordinate into a simple Cartesian coordinate. In the three-dimensional case, a complication occurs due to the non-separable angular kinetic energy operator

$$T_{\theta }=-\frac{\hbar^{2}}{2I}\frac{1}{\;\text{sin}\;\theta }\frac{\partial }{\partial \theta }\text{sin}\;\theta \frac{\partial }{\partial \theta }.$$ (47)

However, as the eigenfunctions (Legendre polynomials) and eigenvalues of this operator are well-known, instead of the Fourier basis representation using the linear momentum eigenfunctions, one may instead employ a basis representation using Legendre polynomials.

Figure 7 depicts the electronic population dynamics induced by the 50 fs FWHM Gaussian pump pulse in the four different models. Although the three results which ignore the nonadiabatic couplings are qualitatively similar, the figure shows that the additional degrees of freedom decelerate the transfer of population from the ground state. The nonadiabatic couplings, which are only present in the three-dimensional model, induce a population transfer from the excited state Q30+ to state Q11 but, on the time scale of the pulse, do not significantly affect the depopulation of the ground state.

**FIG. 8.**
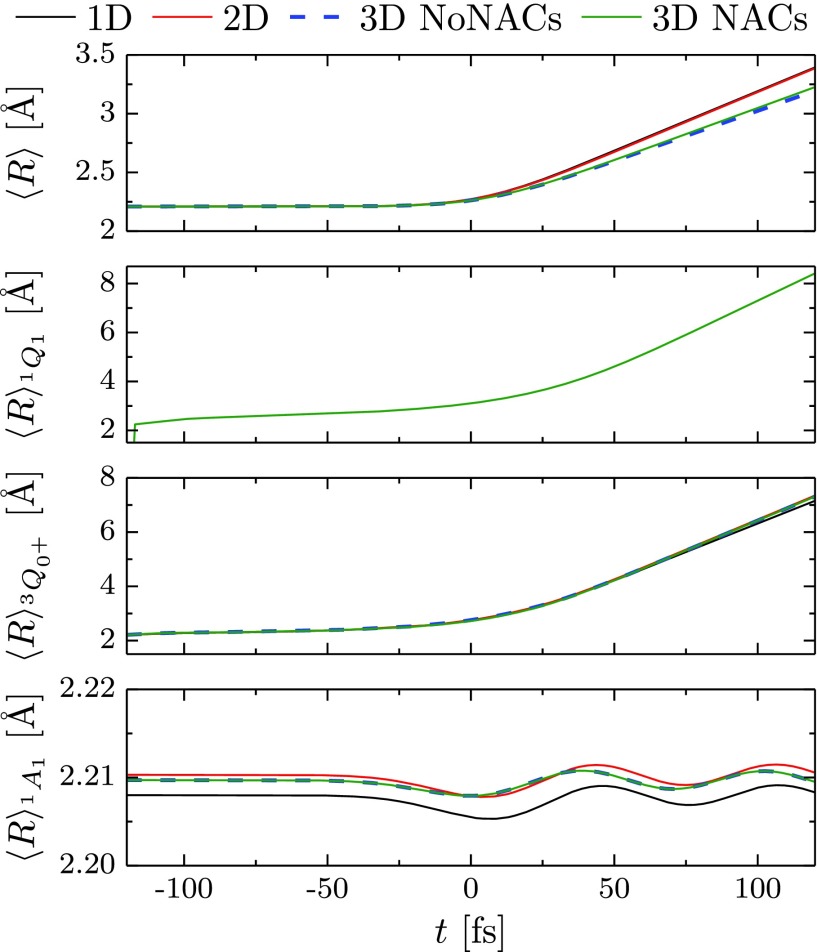
Dissociation dynamics of iodomethane induced by a pump laser pulse. Time-dependence of the expectation value of the dissociative coordinate *R* averaged over the full molecular wavepacket (top panel) and restricted to the vibrational wavepackets in individual electronic states (bottom three panels).

The dissociation dynamics is displayed in Fig. 8, showing the time dependence of expectation value of the dissociative coordinate *R* for the full molecular wavepacket, as well as separately for the nuclear wavepacket on each electronic state. A small difference between the results of the two- and three-dimensional models implies that it is only the angular coordinate *θ* that slightly changes the photodissociation time scale, which is almost unaffected by including the *r* coordinate or nonadiabatic couplings. As expected, the dissociative coordinate *R* plays the leading role in the photodissociation process.

**FIG. 9.**
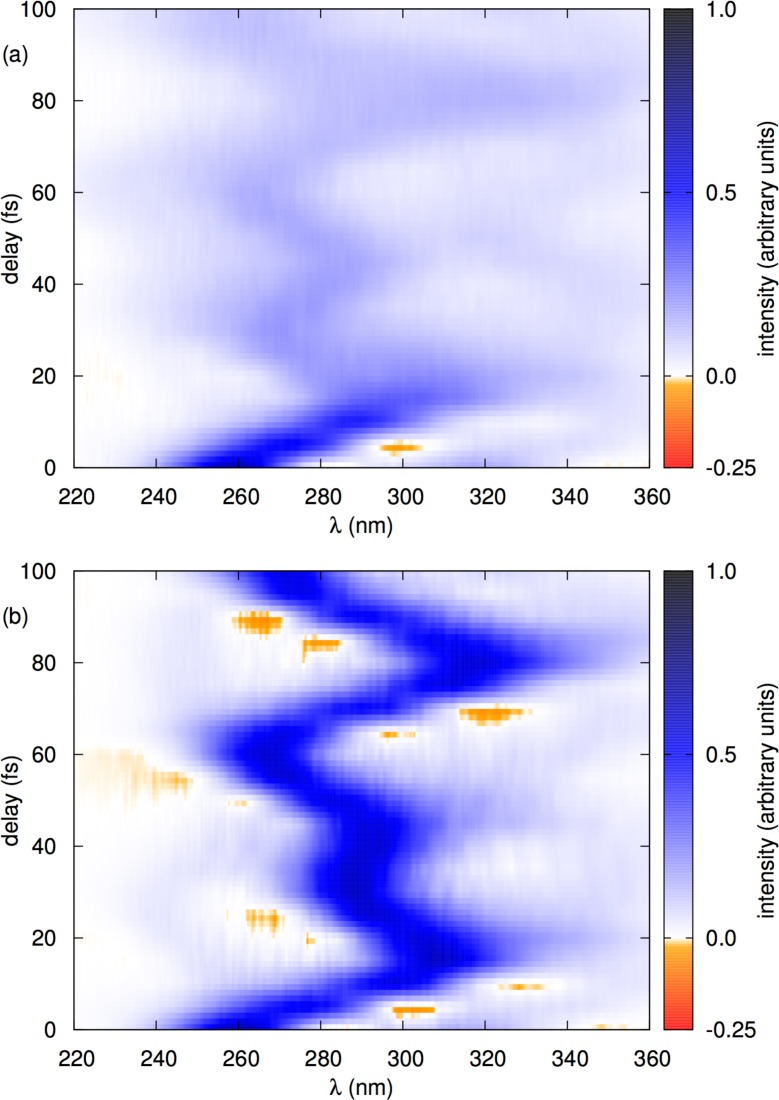
Influence of nonadiabatic dynamics on the time-resolved stimulated emission spectrum of pyrazine. Comparison of numerically evaluated spectra in which the nonadiabatic couplings were included [panel (a)] or neglected [panel (b)]. (a) Due to nonadiabatic dynamics, the signal decays with an increasing delay time *τ*. (b) In the absence of nonadiabatic couplings, the signal does not decay. Both spectra were computed with the MSDR combined with the fewest-switches surface hopping dynamics. Reproduced with permission from Zimmermann and Vaníček, J. Chem. Phys. **141**, 134102 (2014). Copyright 2014 AIP Publishing LLC.

#### 2. Time-resolved stimulated emission of pyrazine studied with adiabatic and nonadiabatic semiclassical dynamics

Vibronic spectroscopy belongs among the most valuable tools for observing the nonadiabatic effects. The effects of nonadiabatic couplings on the linear absorption spectra include line broadening due to the decay of excited state populations by internal conversion or inter-system crossing, intensity borrowing between coupled electronic states, and energy shifts of spectral peaks. The non-linear pump-probe schemes, such as time-resolved stimulated emission, in addition allow to observe some of these effects directly in the time domain.

Nonadiabatic vibronic spectra can be evaluated by a variety of exact quantum, semiclassical, and mixed quantum-classical approaches, including the Multiconfigurational Time-Dependent Hartree (MCTDH) method,^111^ methods employing the hierarchical equations of motion^112–114^ or cumulant expansion,^115^ methods using the semiclassical propagation of Gaussian basis functions,^116,117^ and methods based on the fewest-switches surface hopping or Ehrenfest dynamics,^118–129^ on a single-trajectory dynamics^130^ or on the Fokker-Planck equation,^131^ among many others.

Figure 9 shows the time-resolved stimulated emission spectrum of the S_2_ state of pyrazine computed using models either with or without nonadiabatic couplings. Comparison of the two spectra clearly demonstrates that the decay of the signal on the time scale of tens of femtoseconds may be attributed to nonadiabatic effects, especially to the decay of the S_2_ population to the S_1_ state. The spectrum was computed with the MSDR for nonadiabatic spectra^132^ which is closely related to the MSDR method for estimating adiabaticity,^16^ discussed in Sec. II D, but which instead of fidelity evaluates the dipole-dipole autocorrelation functions semiclassically using the Wigner phase-space formulation of quantum mechanics. The nonadiabatic trajectories are propagated either with the fewest-switches surface hopping or with the locally mean-field dynamics, which is an extension of the Ehrenfest dynamics from a single trajectory to an ensemble of trajectories.^16^

**FIG. 10.**
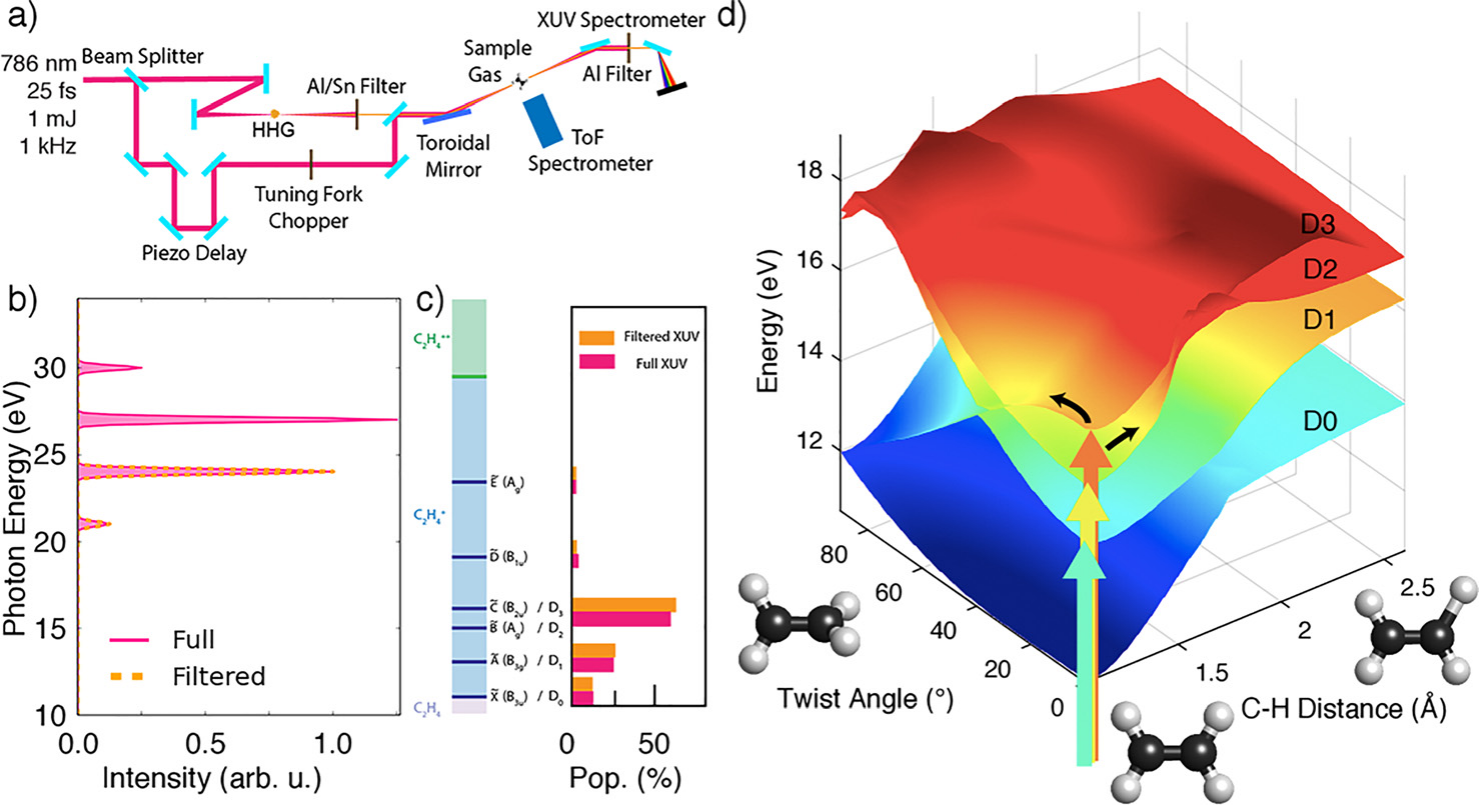
(a) Sketch of the experimental setup: the high order harmonic radiation is recombined with the fundamental IR (probe) pulses through a drilled mirror. (b) extreme ultra violet (XUV) excitation spectrum: a tin filter allows us to switch between the full (solid magenta) and a filtered (dashed orange line) spectrum. (c) Left: cationic states (blue) reachable with the XUV photon energies.^138^ Right: initial population of the cationic states.^138^ (d) D0, D1, D2, and D3 potential energy surfaces along the C-H stretch coordinate and the twist angle, calculated at the aLR-TDDFT/PBE0 level of theory with a 6–31** basis set on the ground-state geometry optimised geometries of the cation. The reference zero energy is the ground state energy of the neutral molecule.

#### 3. Dissociation dynamics of ethylene

Neutral ethylene and its cation have been intensively studied as model systems of a *π*-system and of a *π*-radical.^133–136^

In the pump probe experiment carried out at the attoline of ETH Zurich^137^ [Fig. 10(a)], information on the ethylene cation relaxation dynamics is inferred by recording the delay-dependent ion mass spectra. Attosecond pulse trains, whose spectrum consists of 4 harmonics in the range of 20–30 eV, were used to ionize ethylene molecules, initially in their electronic ground state. Insertion of an Sn filter in front of the extreme ultra violet (XUV) beam allows to reduce the spectrum to the 2 lowest energy harmonics only [Fig. 10(b)]. Due to the photon energies of the pump, different excited states of the $C_{2}H_{2}^{+}$ cation are populated [Fig. 10(c)], while the possibility of double ionization to $C_{2}H_{4}^{++}$ can be ruled out. A probe pulse of photon energy of 1.6 eV, duration of 25 fs and variable intensity was applied, varying the time delay, to probe the dynamics through changes in the measured yield of positively charged fragments.

**FIG. 11.**
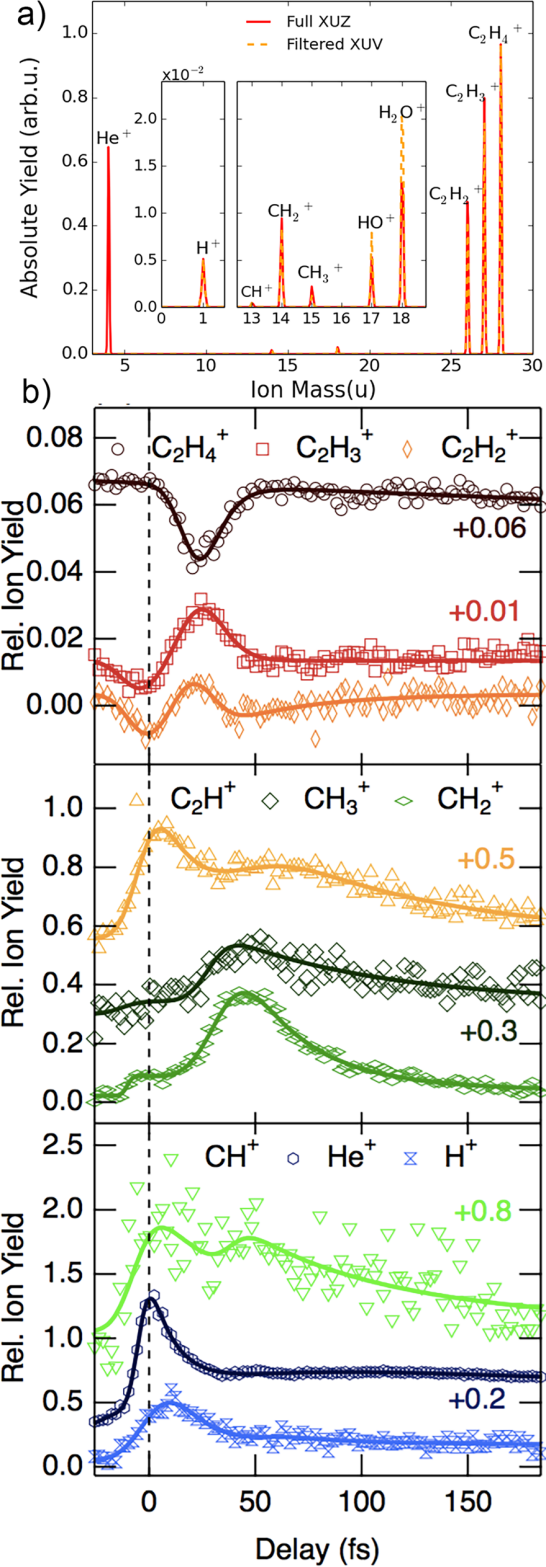
(a) Measured ion spectrum resulting from XUV photoionization of ethylene with the pump pulses of Fig. 1(b). (b) Delay-dependent IR-induced relative charge in the yields. For improved readability, to some curves a vertical offset is applied.

The XUV pump has the effect of bringing the system on the ground or on one of the electronically excited states of the cation: using tabulated values^138,139^ for the cross sections of ethylene for monochromatic light, we estimated that 95% of the electronic state population is confined to the cation ground state and the first three excited states described by the spectroscopic states ${\tilde{X}}^{2}B_{3u},\;{\tilde{A}}^{2}B_{3g},\;{\tilde{B}}^{2}A_{g}$, and ${\tilde{C}}^{2}B_{2u}$ [Fig. 10(d)].

The following dynamics can be represented as the relaxation of the nuclear wave packet across different potential energy surfaces (PES) that eventually brings the system to the cationic ground state or a dissociated state. The final state determines the asymptotic fragment yield, measured from the ion mass spectra and depicted in Fig. 11(a): the dominant fragmentations correspond to the loss of one or two hydrogen atoms, leading to the formation of the $C_{2}H_{3}^{+}$ and $C_{2}H_{2}^{+}$ species. The C-C bond breaking is less frequent and yields of the smaller fragments (${\text{CH}}_{3}^{+},\;{\text{CH}}_{2}^{+},\;{\text{CH}}^{+}$) are at least 100 times smaller. Depending on the pump-probe time delay, the IR probe pulse can alter the way the wave packet relaxes through the various PES, thus changing the fragment yield.

**FIG. 12.**
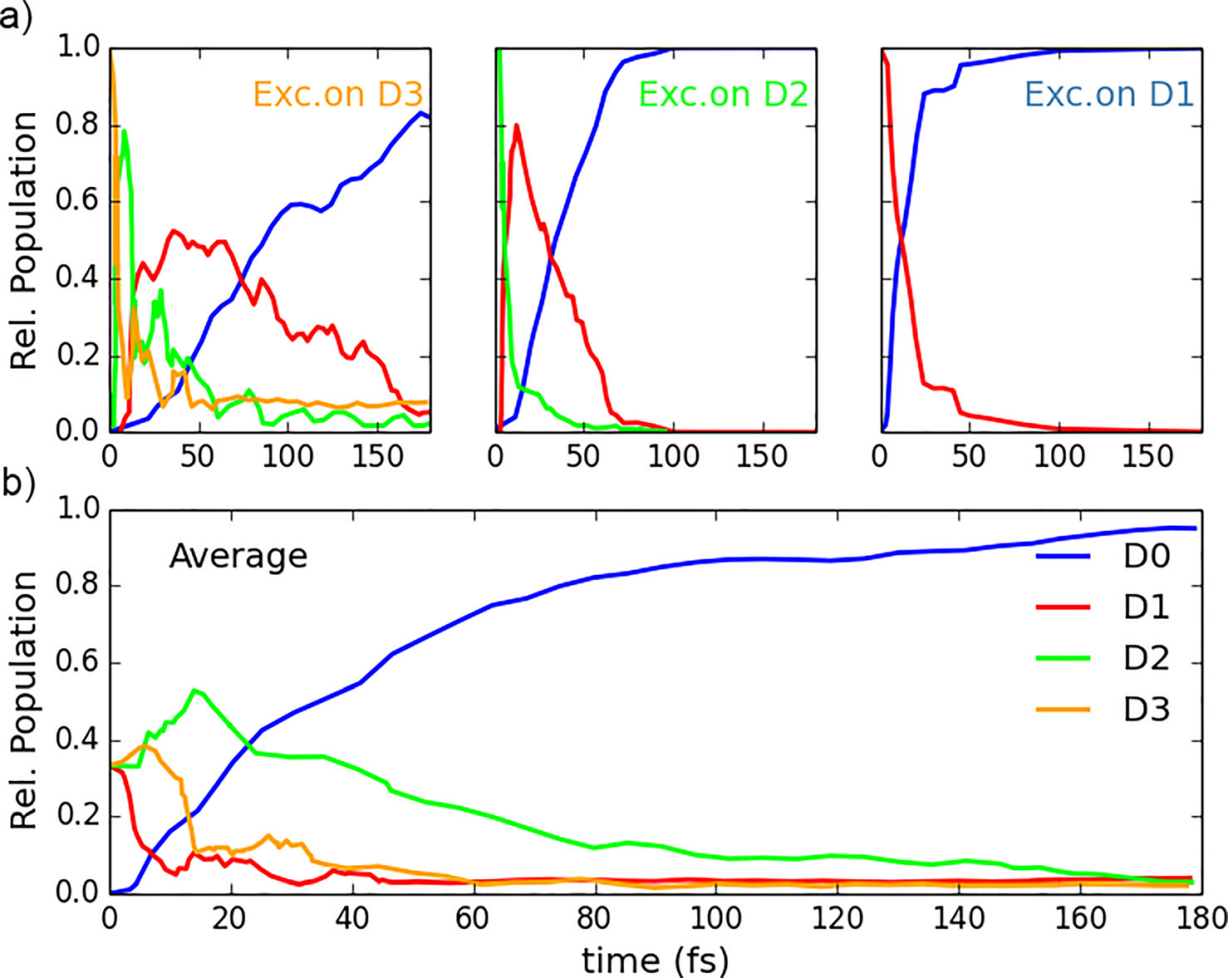
Calculated population of the four lowest cationic states as a function of the time, after selective excitation to D3, D2, and D1 [panel (a)] and averaged over all the initial excitations [panel (b)].

Figure 11(b) shows the IR-induced change in the ion yields relative to the XUV-only yields as a function of the pump-probe delay for an IR peak intensity of 2.5 × 10^1^2 W/cm^2^, a delay step size of 2 fs, and accumulation over 20 000 laser shots per data point. Datapoint fits based on the sum of a minimal number of exponentially modified Gaussian (EMG) functions^140^ are used to extract the delay position of the maximum yield. In all the curves, the characteristic features, confined within the first 60 fs after excitation, remain stable by changing the IR probe intensity, as well as filtering out the two highest harmonic in the XUV spectrum, and are hence representative of the modification to the relaxation dynamics induced by the IR probe.

The maximum yield of the dominant fragmentation channels, $C_{2}H_{3}^{+}$ and $C_{2}H_{2}^{+}$, correlates with the depletion of the cationic species, all occurring at ∼25fs time-delay. Marked yield changes are present also in the curves relative to the monocarbon fragments. The presence of a ${\text{CH}}_{3}^{+}$ fragment is interpreted as the fingerprint of ethylene-ethylidene isomerisation and the position of the ${\text{CH}}_{3}^{+}$ fragment yield maximum (∼40 fs) can be used to extract an upper bound of the isomerisation time, previously estimated to be 50 ± 25 fs.^141^ Thanks to our accurate time-zero calibration, the time resolution and the good statistics of our measurements, considering the instrument response function we obtain a more precise estimate of 30 ± 3 fs.

Trajectory Surface Hopping simulations were performed to gain further insights into the relaxation dynamics of the ethylene cation. The electronic properties (excitation energies, forces and couplings among PES) are obtained in the framework of the Linear Response formulation of the Time-Dependent Density Functional Theory (LR-TDDFT).^47,48^ TSH simulations were carried out with the Newton.X package^142^ interfaced with GAUSSIAN G09. We concentrated our analysis on the four lowest cationic states (D0, D1, D2, and D3), which correspond to the above-mentioned spectroscopic states ${\tilde{X}}^{2}B_{3u}$ and ${\tilde{A}}^{2}B_{3g},\;{\tilde{B}}^{2}A_{g}$, and ${\tilde{C}}^{2}B_{2u}$. We use the 6–31G** basis set on the LR-TDDFT/PBE0 level of theory and 200 TSH trajectories per excited state. Initial geometries and velocities are obtained from Wigner distributions of the neutral ethylene ground state.

Calculation of the cationic PES, as a function of the torsional angle *τ* and one C-H stretch coordinate and of the latter and of the C-C bond distance, evidences the presence of three D1-D0 Conical Intersections (CIs) that can play a role in which of the possible final states ($C_{2}H_{4}^{+}$ ground state, fragmentation) is reached by the system: a twisted CI (*τ* = 90°), a bridged CI, and a C-H elongated (*d_CI_* = 1.7 Å). The elongated and the bridged CI have already been suspected to favour the H^136^ and the H_2_^134,135^ loss.

Additional information can be extracted from the TSH dynamics. The temporal evolution of different state populations is shown in Fig. 12. Our relaxation times are consistent with those found in recent *Ab Initio* Multiple Spawning (AIMS) calculations initialised on the cation geometry rather than on the neutral geometry as in the present work. The majority of the initial population relaxes to the ground state within the first 50 fs after excitation. The averaged population on D1 shows a distinct maximum after ∼15–20 fs, then decays to the ground state.

**FIG. 13.**
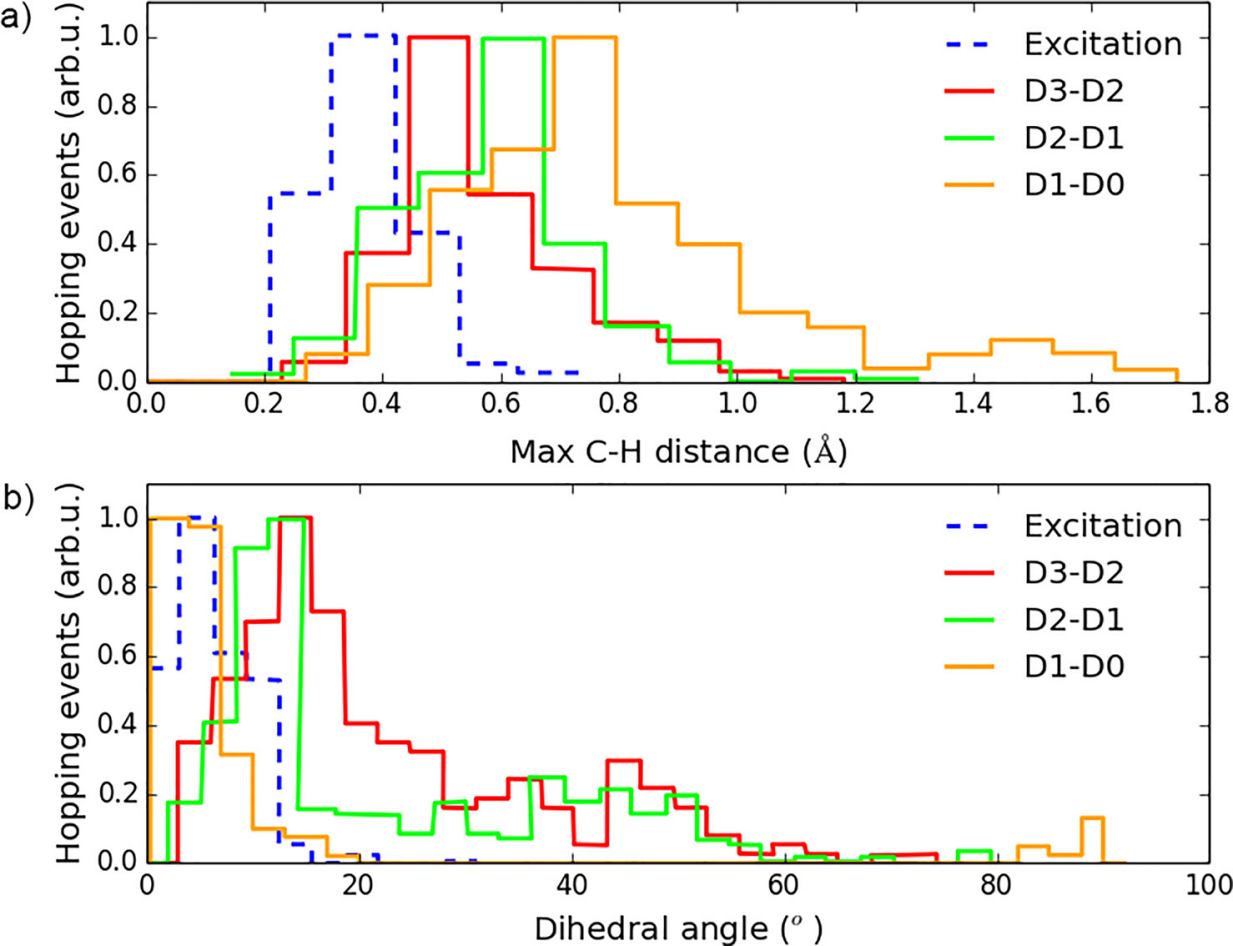
Hopping geometries for transition between different pairs of states. (a) Maximum C-H distance distributions and (b) torsion angle distributions.

The timescale of relaxation to D1 is in a good agreement with the 25 fs delay for the IR-induced change in the main fragmentation channels. Assuming that the branching into different channels occurs around the D1-D0 CIs, the IR pulse probes the presence of the nuclear wave packet around these conical intersections, providing timing information on the relaxation.

Finally, Fig. 13 shows the distributions of C-H stretch coordinates and dihedral angles of all hopping events for transitions between different PESs together with the initial excitation geometry. The tail in the D1/D0 C-H histogram peaked at a C-H distance of ∼1.7 Å and points at the C-H elongated CI, reinforcing the assumption that this CI plays a significant role in the breakup of one C-H bond.

**FIG. 14.**
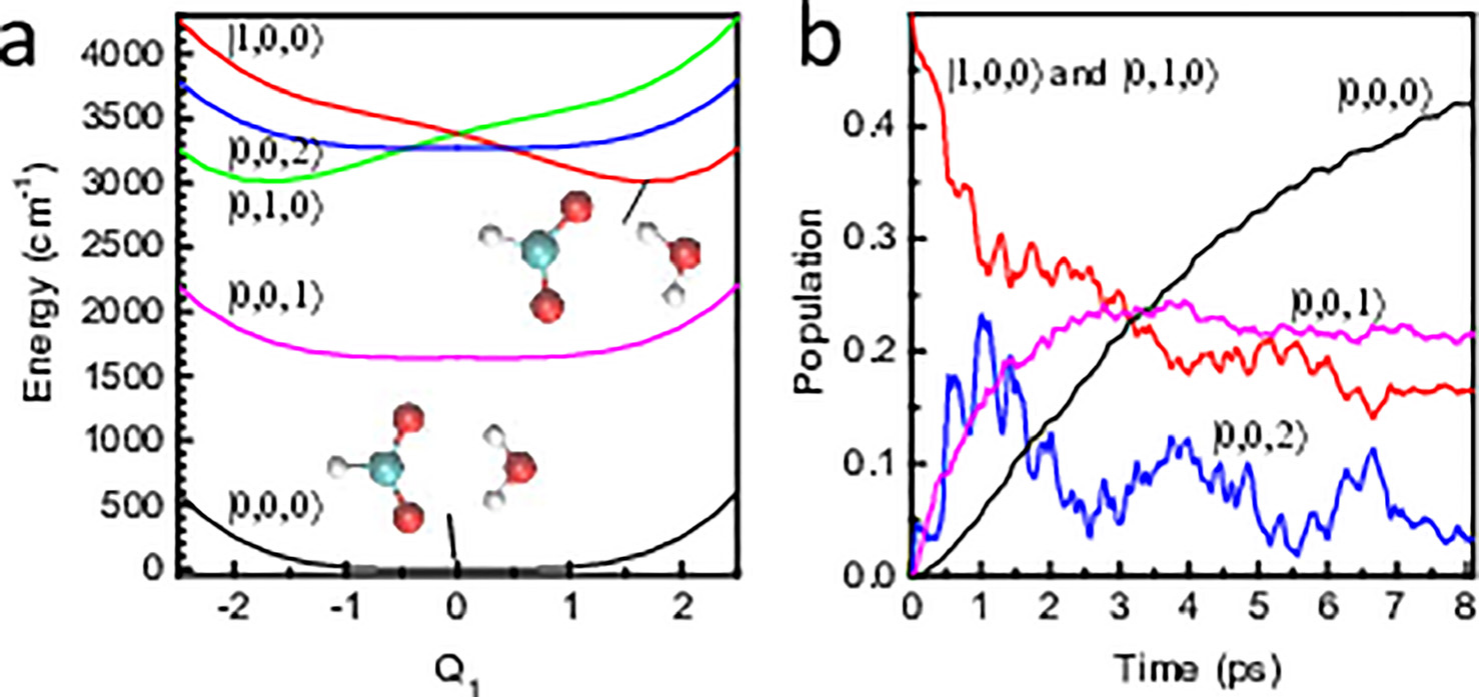
(a) Diabatic potential energy surfaces of the two local OH stretch vibrations of water (|1,0,0⟩ in red and |0,1,0⟩ in green) and the fundamental (|0,0,1⟩ in magenta) and the first overtone of the HOH bending vibration (|0,0,2⟩ in blue) as well as the ground state (|0,0,0⟩ in black). The inter-molecular structures of the complex in the minima of the |0,0,0⟩ and |1,0,0⟩ surfaces are shown. (b) Vibrational relaxation after a “vertical” excitation into the OH stretch modes, using the same color code as in panel (a) for the various states. For symmetry reasons, the population of state |0,1,0⟩ is the same as that of |1,0,0⟩ and is not shown. Adapted with permission from Hamm and Stock J. Chem. Phys. **143**, 134308 (2015). Copyright 2015 AIP Publishing LLC.

#### 4. Ultrafast relaxation of transition metal complexes in solution: Intersystem crossing (ISC)

The group of Chergui *et al.* has been using new methods to probe the nonadiabatic dynamics in molecular systems, predominantly transition metal complexes, and the interplay between charge, spin, and structural dynamics. The methods consist of ultrafast fluorescence up-conversion, 2D UV^143^ and visible spectroscopies,^144^ X-ray spectroscopies,^145^ and photoelectron spectroscopy,^146,147^ all of which were developed in the context of Molecular Ultrafast Science and Technology (MUST), coupled with advanced theoretical simulations.

In a series of studies using fluorescence up-conversion, it was shown that in a large class of organic and inorganic polyatomic molecules, electronic relaxation can proceed at extremely fast time scales, often faster that the highest frequency vibrational modes of the system.^148–153^ Although the systems appear promptly in the lowest electronic states (i.e., electronic cooling is immediate), the energy has been transferred impulsively to low frequency (usually optically silent) modes.

Focusing on the much studied photoinduced spin cross-over (SCO) Δ*S* = 2 transition in Fe(II) complexes, we determined its time scale using 2D UV spectroscopy at a high time resolution and found that it occurs in 50 fs!^154^ This makes it the shortest time scale for the largest spin change ever reported. Our studies on this and other complexes show that at ultrashort time scales, there is no “heavy-atom effect” in intersystem crossing (ISC). Rather, density of states (DOS) and structural dynamics additionally determine the speed of the ISC.^153,155^

Indeed, in the case of diplatinum complexes, the DOS is lower and the ISC rate too, but surprisingly, a strong solvent effect was also observed on the ISC. The reason being that the singlet (S)-triplet (T) transition is favoured by an intermediate state whose energetics are strongly solvent dependent.^156,157^ In some solvents, the acceleration of the ISC is such that a transfer of vibrational coherence was observed from the S to the T state for the first time. Such transfers of vibrational coherence have been rationalized by quantum dynamics simulations in the case of the copper-phenanthroline complex.^158,159^ These simulations were carried out in relation to previous time-resolved X-ray absorption spectroscopy (XAS) experiments,^160^ and the dynamics they unravel have been used as a basis to explore the observation of ultrafast nonadiabatic dynamics by X-ray methods.^161^ Such exploratory simulations have recently been extended to organic molecules and the soft X-ray regime^162,163^ and they are related to the recent work done within MUST by the Wörner and Wolf groups.

Polypyridine complexes of ruthenium(II) have been of major interest within the scientific community due to their relatively low metal-to-ligand charge-transfer (MLCT) states, which readily undergo electron transfer to suitable acceptors. The [Ru(bpy)_3_]^2+^ photophysical properties are strongly influenced by the solvent and thus a thorough understanding of its solvation dynamics is vital to the rationalisation of photochemical behaviour. Moret *et al.*^164^ studied the solvation effects of this cation in the first triplet ^3^MLCT state, using both a hybrid QM/MM Car-Parrinello scheme and classical molecular dynamics. The trajectory analysis described therein revealed that the first solvation sphere consists of chains of approximately 15 H-bonded water molecules which intercalate between the bpy ligands, inducing a secondary solvation sphere. Consequently, water residence time within the first solvation shell was computed to be 12.4 ps, which is significantly larger than the value of 4.5 ps computed for bulk water. These observations support the experimentally observed “tight association” of water molecules to the [Ru(bpy)_3_]^2+^ complex. Furthermore, it was found that the dipole of water molecules can easily reorientate due to the one-dimensional arrangement of hydrogen bonds, which enables an efficient and fast stabilisation of the ^3^MLCT state induced by photoexcitation.

The extensive use of group 8 metals as chromophores in light-harvesting dendritic polymers and sensitisation of wide-band gap semiconductors exemplifies the importance of understanding the electron dynamics following a photoexcitation. The doorway state to charge separation in [Ru(bpy)_3_]^2+^ is first its lowest singlet ^1^MLCT excited state. From here, it is known to undergo an intersystem crossing (ISC) to a long-lived triplet state (^3^MLCT) with unity quantum efficiency. However, despite the enormous importance of this photophysical process, there is still a lack of understanding on the localisation dynamics of the resulting photoexcited electron in the ligand system. Moret *et al.*^165^ used hybrid QM/MM Car-Parrinello molecular dynamics to study the solvent-mediated electron localization and dynamics in the long-lived ^3^MLCT state. In the gas phase, the unpaired electron is found to be delocalized over all three bipyridine units. In contrast, the simulations of the water-solvated complex revealed a thermal-relaxation of the complex and solvent-induced break of the coordination symmetry. This drives a spin-density localization of the photoexcited electron onto predominantly two of the bipyridine units, contrary to the generally accepted idea of localization onto a single ligand. The low-energy barrier spin density fluctuations among the ligands constituting this pair were determined to show a characteristic time of approximately 0.5–1.0 ps. This indicates that a simple interligand electron transfer (ILET) mechanism involving well localized ligand orbitals does not adequately describe the photoexcitation process.

LR-TDDFT based surface hopping QM/MM simulations^166–168^ were applied to study the ultrafast relaxation of the photoexcited singlet ^1^MLCT state of [Ru(bpy)_3_]^2+^ solvated in water. Previous experiments have shown that after light irradiation, the complex relaxes within 100 fs from a singlet ^1^MLCT to the long-lived triplet ^3^MLCT state, where it can be observed for several hundred picoseconds. Indeed, the work summarised above focussed on the dynamics of the long-lived triplet state, whereas here, the first steps of this photophysical process are of interest, characterized by the nonadiabatic relaxation of the excited ^1^MLCT state and its crossing with the lower lying ^3^MLCT states. The spin-orbit coupling t elements for the singlet-triplet crossing were qualitatively estimated based on an analysis of the assignments of the excited states involved, justified by the quantum yield of the ^1^MLCT-^3^MLCT ISC being close to unity. By performing nonadiabatic dynamics from the first ^1^MLCT with large oscillator strength, such an approximation uncovered that several intersystem crossing events can readily occur within 40–50 fs of dynamics, in good agreement with the experiment.

The [Ru(bpy)_3_]^2+^ complex (where bpy = 2,2′-bipyridine or analogous *α*-diimines) is particularly redox reactive in solution and readily participates in electron transfer reactions. The singlet ground state of [Ru(bpy)_3_]^2+^ is well characterized in this regard, with a well-established oxidation potential of 1.26 V vs. the standard hydrogen electrode (SHE). Conversely, the redox activity of the triplet state was not well understood, and while the oxidization potential had been estimated to be −0.86 V vs. the SHE, this was under the assumption that structural changes and reorganisation in transitions between the triplet and singlet states are negligible. Diamantis *et al.*,^169^ computed the redox properties of [Ru(bpy)_3_]^2+^ in both its ^1^MLCT singlet and ^3^MLCT triplet states at the DFT/BP86 level. Here, the authors also attempt to quantify the inaccuracy induced by using a QM/MM model vs. a full QM treatment, as standard QM/MM approaches do not include polarization of the MM region by the QM region, which would require a polarizable force field. Indeed, this work found that QM/MM simulations can predict Helmholtz free energy changes for redox half-reactions that are in good agreement with values obtained from a full QM treatment. Additionally, the authors found that the entropy change during a singlet-triplet transition is 0.27 eV, which disagrees with a previously assumed estimate of 0.03 eV leading to an oxidization potential of −0.62 V for triplet [Ru(bpy)_3_]^2+^.

The [ReX(CO)_3_(bpy)]^*n*+^ family of complexes, where X = halide or pseudohalide are notable examples of metal complexes that exhibit complex electronic structure features—their valence space is composed of *π*-electron accepting CO and bpy ligands, with a *π*-electron donating (pseudo)halide. The low-lying excited states for these complexes are expected to exhibit a Re → bpy and X → bpy charge transfer (CT) character, which has led to them being labelled as metal-ligand-to-ligand charge transfer (MLLCT) states. However, direct experimental evidence of this two-center transfer was previously lacking. In a work by Nahhas *et al.*,^170^ static and picosecond X-ray absorption spectroscopy (XAS) was used to probe the electronic and geometric structure of ground and excited [ReX(CO)_3_(bpy)]^*n*+^ (X = Etpy; n = 1, Cl, or Br; n = 0). Additionally, DFT calculations were performed for the ground and excited states to reproduce experimental X-ray absorption near edge (XANES) and extended X-ray absorption fine structure (EXAFS) spectra, with an accurate reproduction of the Re L_3_-edge. When X = Br, the K-edge spectrum shows a strong solvent effect, due to the direct exposure of the Br ligand to the environment. Additionally, the transient spectra at both the Re L_3_ and Br K-edges display a pre-edge feature not observed in the ground state spectrum, and are attributed to electron depletion from the HOMO following a photoexcitation. These features have very similar dynamics, which confirms previous predictions that the low-lying states involve charge transfer from both Re and X ligand, hence, are best described as MLLCT states. In another work, Penfold *et al.*^171^ present a wavelet transform analysis for the x-ray absorption spectra of molecules, which yields a 2D correlation plot in both *R*- and *k*-space. This permits the unambiguous assignment of fine structure oscillations for complex systems. This was applied to both iron hexacyanide in both its ferric and ferrous form, and the rhenium diimine complex mentioned previously.

Related work investigating nonadiabatic effects in transition metal complexes can be found in Refs. 160 and 172–176.

### C. Nonadiabatic effects in vibrational dynamics

**FIG. 15.**
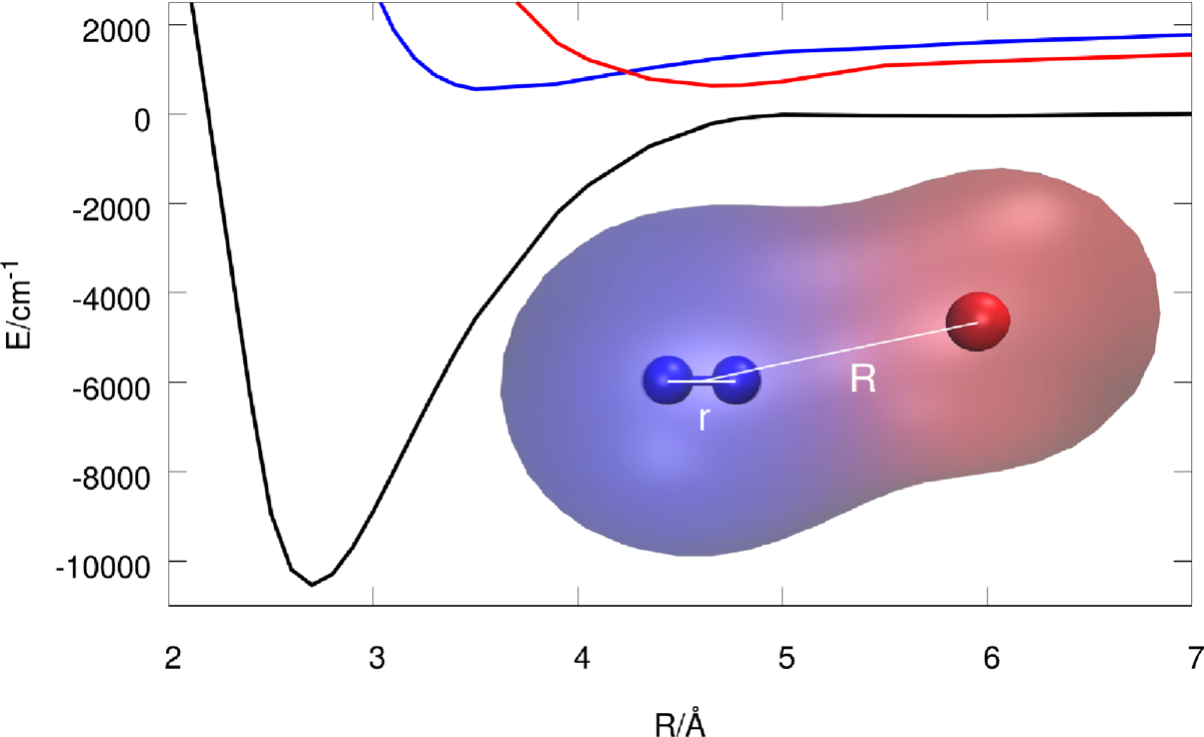
(a) Interaction energy at the inner turning point (*r* = 1.072 Å) of the *v* = 0 vibration for the three lowest electronic states of (N_2_^+^-Ar) (the angle between *r* and *R* is 168°). The curves were computed at the MRCI+Q/avtz level of theory in the ^2^A' symmetry. Note that the separation between ground state and first excited state is only about 700 cm^−1^ at around *R* = 4.5 Å.

Also for purely vibrational problems in the electronic ground state, an adiabatic separation of two sets of vibrational degrees of freedom is frequently applied,^176–185^ which leads to very similar equations and pictures as the Born-Oppenheimer approximation for coupled electronic-vibrational problems. Such an adiabatic separation is most meaningful when considering high-frequency intramolecular vibrational modes of a molecule vs. low-frequency intermolecular degrees of freedom. That situation occurs, for example, when two molecules dimerize via hydrogen bonds or when a particular molecular group is solvated. When both high-frequency and low frequency degrees of freedom are coupled, an adiabatic separation leads to the picture of potential energy surfaces of the various vibrational quantum states of the high-frequency modes as a function of inter-molecular configuration [Fig. 15(a)]. In hydrogen-bonded complexes, the coupling between intra- and intermolecular degrees of freedom is particularly large, hence, the tuning of those quantum states is large, so that they will likely cross and also form vibrational conical intersections^186,187^ (vibrational conical intersections have also been identified in other molecular systems^184,185^). In the electronic case, conical intersections are the hallmark of ultrafast photophysics and photochemistry.^188^ One may therefore assume that the same is true in the vibrational case, i.e., that vibrational conical intersections are responsible for the often observed very fast (100 fs) vibrational relaxation in hydrogen bonded systems^189–191^ together with sometimes very complex oscillatory features.^192–195^ Furthermore, the typical dissociation energy of a hydrogen bond is in the same range as the intramolecular frequency of the participating OH stretch vibration and speculations about IR induced photochemistry, i.e., hydrogen-bond breaking, appeared in literature.^176,177,196^

These ideas have been made explicit in Ref. 3, where we studied vibrational conical intersections and the quality of the adiabatic picture for the hydrogen-bonded ${\text{HCO}}_{2}^{-}\cdot H_{2}O$ complex. That molecular system has been studied extensively in the gas-phase by Johnson and coworkers, revealing very peculiar IR spectra of the OH-stretch vibration of water.^178,183,197^ Figure 14(a) shows the potential energy surfaces of the two local OH stretch vibrations and the bending vibration of water as a function of the water rocking mode *Q*_1_, which is the inter-molecular degree of freedom that tunes the energies of the high-frequency vibrational states the most. It can be seen that the two OH stretch vibrations (shown in red and green) and the first overtone of the HOH bending vibration (shown in blue) have numerous crossings [note that Fig. 14(a) shows the diabatic potential energy surfaces], which indeed evolve into conical intersections when considering the full dimensionality of the inter-molecular degrees of freedom of the system.^3^ Based on these potential energy surfaces, the vibrational energy relaxation pathways have been calculated after “vertical” excitation into the OH stretch modes (that carry the oscillator strength), implicitly including all nonadiabatic couplings (see Fig. 14). Somewhat surprisingly, the build-up of population of the first overtone of the HOH bending vibration (|0,0,2⟩, shown in blue) is not faster than that of its fundamental (|0,0,1⟩, shown in magenta), despite the fact that the latter is not connected to the initially pumped OH stretch modes by any conical intersections. Hence, different to the electronic case, vibrational conical intersections do not seem to be distinguished pathways of energy flow, suggesting that the adiabatic approximation is in fact relatively poor to start with.

This finding might appear surprising in the light of the fact that the timescale separation of inter- and intramolecular degrees of freedom in the purely vibrational case is quite similar to that of vibrational and electronic degrees of freedom in the electronic case; a factor of 10–20 in both cases. In a very sloppy way, it is often said that it is that timescale separation, which is the condition for the adiabatic approximation to be good. However, a close inspection of Eqs. (9) and (14) reveals that it is really the mass difference of the electron and nuclei that renders the Born-Oppenheimer approximation good, and not the timescale separation *per se*; the timescale separation is only a consequence of the mass difference. In the electronic case, the mass ratio is $m_{el}/m_{nuc}\approx 10^{-4}–10^{-5}$, while the ratio of reduced masses of high- vs. low frequency modes is only ≈10^−1^ in the purely vibrational case. The timescale of a particular degree of freedom, in turn, scales as $\sqrt{m/k}$ (assuming a harmonic picture with *k* being a force constant). In the electronic case with a mass ratio of ≈ 10^−4^–10^−5^, the potentials are such that they actually reduce the timescale separation from what would expected from a simple $\sqrt{m}$ dependence. In contrast in the vibrational case, both the relatively small mass ratio and the potentials act in concert—intermolecular forces are weaker than intramolecular forces—to reveal about the same timescale separation as in the electronic case.

### D. Nonadiabatic effects in ion-atom collisions

The collision of N_2_^+^ ions with Ar atoms is a prototypical system to study ion-atom collisions and charge transfer reactions. It might also be an example of a system where nonadiabatic effects are important in order to understand the underlying dynamics. Previous experiments by Schlemmer *et al.*^198^ on this system have uncovered an unusual behaviour regarding the rotational relaxation of N_2_^+^ ions. Since the (_2_^+^-Ar) complex has a large binding energy of 1.109 eV,^199^ collisions at low temperatures should proceed via a long-lived bound state. Since this usually leads to statistical mixing of all accessible product channels, a quick redistribution of rotational states can be expected. However, Schlemmer *et al.* found a very low rate coefficient of *kJ* (90 K) = (1.4 ± 0.4) × 10^11^ cm^3^s^−1^ for rotational relaxation, which implies that the contrary is true. Assuming that collisions occur at the Langevin rate *k_L_* = 7.4 × 10^−10^ cm^3^s^−1^ , this implies that the rotational state of N_2_^+^ ions is conserved for about 49 out of 50 collisions with Ar atoms (according to the Langevin model, a collision complex is formed if the collision energy is sufficient to overcome the rotational barrier).^198^

Schlemmer *et al.* proposed that this unexpected result could either be explained as the consequence of some hidden constants of motion leading to approximate selection rules, or by the presence of additional barriers in the (N_2_^+^-Ar) potential energy surface (PES). They remark that “detailed *ab initio* calculations and quantum mechanical treatment of the collision dynamics at meV energies are required to conclusively answer the question.”^198^

Meuwly and co-workers^200,201^ constructed a state of the art (N_2_^+^-Ar) PES based on *ab initio* energies computed at the UCCSD(T)-F12a/aug-cc-VTZ level of theory using reproducing kernel Hilbert space (RKHS) theory.^202,203^ The RKHS method has the appealing quality that it exactly reproduces the given data, therefore allowing energy evaluations at practically ab initio quality.^200^ In order to assess the importance of multireference effects, additional calculations at the CASSCF/MRCI+Q level of theory were performed and it was concluded that UCCSD(T)-F12a/aug-cc-VTZ is a sufficiently high level of theory.^201^ Quantum close-coupling calculations^201^ as well as quasi-classical trajectory (QCT) calculations^201^ were performed on the RKHS-PES using conditions similar to those in the experiment by Schlemmer *et al.*^198^ The rate coefficients for rotational relaxation obtained by the quantum and QCT calculations agree favourably and range between 5.34 × 10^−10^ cm^3^s^−1^ and 6.96 × 10^−10^ cm^3^s^−1^.^201^ These rates are in line with the expected quick redistribution of rotational states and suggest that more than 70% of collisions lead to a change of the rotational quantum state.

Due to the high quality of the PES and the agreement of quasi-classical and quantum results, it is unlikely that the discrepancy to the experimentally observed rate is due to deficiencies of the applied computational methods. However, it is possible that the Born-Oppenheimer approximation is not valid for the (N_2_^+^-Ar) complex and nonadiabatic effects are important in the dynamics. This assumption is supported by the observation that the lowest excited electronic state of (N_2_^+^-Ar) is only energetically separated by about 700 cm^−1^ from the ground state in the long range part of the N_2_^+^+Ar interaction (Fig. 15). In fact, the separation of the electronic states is a function of the vibrational motion that N_2_^+^ undergoes and therefore suggests that a coupling between nuclear and electronic degrees of freedom might exist.^201^

## V. SUMMARY AND OUTLOOK

In this review, we have given an overview of a palette of nonadiabatic phenomena that range from the nonadiabatic electron dynamics observed in time-dependent external fields, to nonadiabatic processes in coupled electron-nuclear dynamics, to a generalization of the nonadiabatic concept to vibrational mode coupling. Although the examples we give originate from very different fields, they share the intrinsic nature of nonadiabaticity induced by a time-dependent change in systems with relatively slow degrees of freedom. As such, several of the theoretical approaches that are discussed herein are general and can be applied to nonadiabatic phenomena of different types or even motivate an extension of these methods to processes (such as vibrational mode coupling) for which they are not routinely applied.
